# Model Projections in Model Space: A Geometric Interpretation of the AIC Allows Estimating the Distance Between Truth and Approximating Models

**DOI:** 10.3389/fevo.2019.00413

**Published:** 2019-11-08

**Authors:** José Miguel Ponciano, Mark L. Taper

**Affiliations:** 1 Biology Department, University of Florida, Gainesville, FL, United States,; 2 Department of Ecology, Montana State University, Bozeman, MT, United States

**Keywords:** error rates in model selection, Kullback-Leibler divergence, model projections, model averaging, Akaike’s Information Criterion

## Abstract

Information criteria have had a profound impact on modern ecological science. They allow researchers to estimate which probabilistic approximating models are closest to the generating process. Unfortunately, information criterion comparison does not tell how good the best model is. In this work, we show that this shortcoming can be resolved by extending the geometric interpretation of Hirotugu Akaike’s original work. Standard information criterion analysis considers only the divergences of each model from the generating process. It is ignored that there are also estimable divergence relationships amongst all of the approximating models. We then show that using both sets of divergences and an estimator of the negative self entropy, a model space can be constructed that includes an estimated location for the generating process. Thus, not only can an analyst determine which model is closest to the generating process, she/he can also determine how close to the generating process the best approximating model is. Properties of the generating process estimated from these projections are more accurate than those estimated by model averaging. We illustrate in detail our findings and our methods with two ecological examples for which we use and test two different neg-selfentropy estimators. The applications of our proposed model projection in model space extend to all areas of science where model selection through information criteria is done.

## 1. INTRODUCTION

Recent decades have witnessed a remarkable growth of statistical ecology as a discipline, and today, stochastic models of complex ecological processes are the hallmark of the most salient publications in ecology (e.g., Leibold et al., 2004; Gravel et al., 2016; Zeng and Rodrigo, 2018). Entropy and the Kullback-Liebler divergence as instruments of scientific inquiry are now at the forefront of the toolbox of quantitative ecologists, and many exciting new opportunities for their use are constantly being proposed (e.g., Casquilho and Rego, 2017; Fan et al., 2017; Kuricheva et al., 2017; Milne and Gupta, 2017; Roach et al., 2017; Cushman, 2018). One of the most important, but under explored, applications of the Kullback-Liebler divergence remains the study or characterization of the error rates incurred while making model selection according to information criteria (Taper and Ponciano, 2016b). This research is particularly relevant when, as it almost always happens in science, none of the candidate models exactly corresponds to the chance mechanism generating the data.

Understanding the impact of misspecification of statistical models constitutes a key knowledge gap in statistical ecology, and many other areas of biological research for that matter (e.g., Yang and Zhu, 2018). Research by us and many others (see citations in Taper and Ponciano, 2016b and in Dennis et al., 2019) has led to detailed characterizations of how the probability of making the wrong model choice using any given information criterion, not only may depend on the amount of information (i.e., sample size) available, but also on the degree of model misspecification.

Consequently, in order to estimate the error rates of model selection according to any information criterion, practitioners are left with the apparent paradox (“catch-22”) of being able to estimate how likely it is to erroneously deem as best that model which is furthest apart from the generating model, only after having accomplished the unsolved task of estimating the location of the candidate models relative to the generating process and to each other.

In this paper, we propose a solution to this problem. Our solution was motivated by the conceptualization of models as objects in a multi-dimensional space as well as an extension of the geometrical thinking that Akaike used so brilliantly in his 1973 paper introducing the AIC. Starting from Akaike’s geometry, we show how to construct a model space that includes not only the set of candidate models but also an estimated location for the generating process. Now, not only can an analyst determine which model is closest to the generating process, she/he can also determine the (hyper)spatial relationships of all models and how close to the generating process the best model is.

In 1973, Hirotugu Akaike wrote a truly seminal paper presenting what came to be known as the AIC. Akaike initially called the statistic “An Information Criterion,” but soon after its publication it came to be known as “Akaike’s Information Criterion.” Various technical accounts deriving the AIC exist (e.g., Burnham and Anderson, 2004, Chapter 7), but few explain in detail every single step of the mathematics of Akaike’s derivation (but see De Leeuw, 1992). Although focusing on the measure-theoretic details, deLeeuw’s account makes it clear that Akaike’s paper was a paper about ideas, more than a paper about a particular technique. Years of research on this project has led us to understand that only after articulating Akaike’s ideas, the direction of a natural extension of his work is easily revealed and understood. Although thinking of models and the generating mechanism as objects with a specific location in space is mathematically challenging, this exercise may also prove to be of use to study the adequacy of another common statistical practice in multi-model inference: model averaging.

Intuitively, if one thinks of the candidate models as a cloud of points in a Euclidean space, then it would only make sense to “average” the model predictions if the best approximation of the generating chance mechanism in that space is located somewhere inside the cloud of models. If however the generating model is located outside such cloud, then performing model average will only at best, worsen the predictions of the closest models to the generating mechanism. The question then is, can this idea of thinking about models as points in a given space be mathematically formalized? Can the structure and location of the candidate models and the generating mechanism be somehow estimated and placed in a space? If so, then the answer to both questions above (i.e., the error rates of multi-model selection under misspecification and when should an analyst perform model averaging) could be readily explored. These questions are the main motivation behind the work presented here.

## 2. THE AIC AND A NATURAL GEOMETRIC EXTENSION: MODEL PROJECTIONS IN MODEL SPACE

In his introduction to Akaike (1973)’s original paper, De Leeuw (1992) insisted on making sure it was understood that Akaike’s contribution was much more valuable for its ideas than for its technical mathematical developments: “…This is an ‘ideas’ paper,’ promoting a new approach to statistics, not a mathematics paper concerned with the detailed properties of a particular technique…” After this explanation, De Leeuw undertakes the difficult labor of teasing Akaike’s thought process from the measure-theoretic techniques. In so doing, the author manages to present a clear and concise account clarifying both, Akaike’s mathematical approach and his ideas. De Leeuw was keenly aware of the difficulty of trying to separate the ideas from the mathematical aspects of the paper: in introducing the key section in Akaike’s paper, he describes it as “a section not particularly easy to read, that does not have the usual proof/theorem format, expansions are given without precise regularity conditions, exact and asymptotic identities are freely mixed, stochastic and deterministic expressions are not clearly distinguished and there are some unfortunate notational… typesetting choices” (De Leeuw, 1992). To us, however, the importance of De Leeuw’s account stems from the fact that it truly brings home the crucial point that at the very heart of Akaike’s derivation there was a geometrical use of Pythagoras’ theorem (see Equation 1, page 604 in De Leeuw, 1992). The modern literature has been able to reduce Akaike’s derivation to just a few lines (see Davison, 2003). However, such condensed proofs conceal the original geometric underpinnings of Akaike’s thinking, which De Leeuw exposed. Our contribution for this special issue consists of taking Akaike’s derivation one step further by using Pythagoras’ theorem again to attain not a relative, but an absolute measure of how close each model in a model set is from the generating process.

Akaike’s (1973) paper is difficult and technical but at the same time, it is a delightful reading because he managed to present his information criterion as the natural consequence of a logical narrative. That logical narrative consisted of six key insights that we strung together to arrive at what we believe is a second natural consequence of Akaike’s foundational thoughts: our model projections proposal. After introducing our notation following Akaike’s, we summarize those six key insights. We stress that these insights and the accompanying key figure we present below are none other than a simple geometric representation of De Leeuw’s measure-theoretic re-writing of Akaike’s proof. We encourage readers with a strong probability background to read De Leeuw’s account. We then present our main model projections proposal and contribution and support it with a fully illustrated example.

### 2.1. Theoretical Insights From Akaike (1973)

Akaike’s quest was motivated by a central goal of modern scientific practice: obtaining a comparison measure between many approximating models and the data-generating process. Akaike began thinking about how to characterize the discrepancy between any given approximating model and the generating process. He denoted the probability densities of the generating process and of the approximating model as *f* (*x*, *θ*_0_) and *f* (*x*, *θ*), respectively, where *θ*_0_ denoted the column vector of dimension *L* of true parameter values. Although he started by characterizing the discrepancy between the true model and the approximating model, his objective was to come up with an estimate of such discrepancy that somehow was free of the need of knowing either the dimension or the model form of *f* (*x*, *θ*_0_). The fact that he was able to come up with an answer to such problem is not only outstanding, but the reason why the usage of the AIC has become ubiquitous in science. Akaike’s series of arguments arriving to the AIC can be summarized by stringing together these six key insights:

#### 2.1.1. Insight 1: Discrepancy From the Generating Process (Truth) Can Be Measured by the Average of Some Function of the Likelihood Ratio

Akaike’s first important insight follows from two observations. First, under the parametric setting defined above, a direct comparison between an approximating model and the true, generating stochastic process can be achieved *via* the likelihood ratio, or some function of the likelihood ratio. Second, because the data *X* are random, the expected discrepancy (average over all possible realizations of the data) would be written as

$$\begin{array}{ll} D\left(\theta ,\theta_{0};\Phi \right) & =\int f\left(x;\theta_{0}\right)\Phi \left(\tau \left(x,\theta ,\theta_{0}\right)\right)dx\\ \; & =E_{X}\left[\Phi \left(\tau \left(X,\theta ,\theta_{0}\right)\right)\right], \end{array}$$

where the expectation is, of course, taken with respect to the generating stochastic process *X*. We denote the likelihood ratio as $\tau \left(x,\theta ,\theta_{0}\right)=\frac{f(x;\theta )}{f\left(x;\theta_{0}\right)}$ and a twice differentiable function of it as Φ(*τ*(*x*, *θ*, *θ*_0_)).

Akaike then proposed to study under a general framework how sensitive this average discrepancy would be to the deviation of *θ* from the truth, *θ*_0_.

#### 2.1.2. Insight 2: $D\left(\theta ,\theta_{0};\Phi \right)$ Is Scaled by Fisher’s Information Matrix

Akaike thought of expanding the average discrepancy $D\left(\theta ,\theta_{0};\Phi \right)$ using a second order series approximation around *θ*_0_. Akaike’s second insight then consisted of noting the strong link between such approximation and the theory of Maximum Likelihood (ML).

For a univariate *θ*, the Taylor series approximation of the average function Φ of the likelihood ratio is written as

(1) $$D\left(\theta ,\theta_{0};\Phi \right)\approx D\left(\theta_{0},\theta_{0};\Phi \right)+{\left(\theta -\theta_{0}\right)\frac{\partial D\left(\theta ,\theta_{0};\Phi \right)}{\partial \theta }|}_{\theta =\theta_{0}}\;+{\frac{{\left(\theta -\theta_{0}\right)}^{2}}{2!}\frac{\partial^{2}D\left(\theta ,\theta_{0};\Phi \right)}{\partial \theta^{2}}|}_{\theta =\theta_{0}}+\ldots$$

To find an interpretable form of this approximation, just like Akaike did following Kullback and Leibler (Kullback and Leibler, 1951; Akaike, 1973), we use two facts: first, by definition *τ*(*x*, *θ*, *θ*_0_)|_*θ*=*θ*0_ = 1 and second, that *∫ f* (*x*; *θ*)*dx* = 1 because *f* is a probability density function. Together with the well-known regularity conditions used in mathematical statistics that allow differentiation under the integral sign (Pawitan, 2001), these two facts give us the following: first, $\int \frac{\partial f(x;\theta )}{\partial \theta }dx=\int \frac{\partial^{2}f(x;\theta )}{\partial \theta^{2}}dx=0$. Hence, ${\frac{\partial D\left(\theta ,\theta_{0};\Phi \right)}{\partial \theta }|}_{\theta =\theta_{0}}=0$. This result then allows writing the second derivative of the approximation as

$$\begin{array}{ll} {\frac{\partial^{2}D\left(\theta ,\theta_{0};\Phi \right)}{\partial \theta^{2}}|}_{\theta =\theta_{0}} & ={\int \frac{\partial }{\partial \theta }\left(\frac{\partial \Phi (\tau )}{\partial \tau }\frac{\partial \tau }{\partial \theta }\right)f\left(x;\theta_{0}\right)dx|}_{\theta =\theta_{0}}\\ & ={\int \frac{\partial^{2}\Phi (\tau )}{\partial \tau^{2}}{\left(\frac{\partial \tau }{\partial \theta }\right)}^{2}f\left(x;\theta_{0}\right)dx|}_{\theta =\theta_{0}}\;+{\int \frac{\partial^{2}\tau }{\partial \theta^{2}}\frac{\partial \Phi (\tau )}{\partial \tau }f\left(x;\theta_{0}\right)dx|}_{\theta =\theta_{0}}\\ & =\Phi^{\prime \prime }(1)\int {\left(\frac{1}{f\left(x;\theta_{0}\right)}\frac{\partial f(x;\theta )}{\partial \theta }\right)}^{2}f\left(x;\theta_{0}\right)dx|S_{\theta =\theta_{0}}\\ & ={\Phi^{\prime \prime }(1)\int {\left(\frac{\partial \text{log}f(x;\theta )}{\partial \theta }\right)}^{2}f(x;\theta )dx|}_{\theta =\theta_{0}}\\ & =\Phi^{\prime \prime }(1)I\left(\theta_{0}\right), \end{array}$$

where $I\left(\theta_{0}\right)$ is Fisher’s information. To move from the first line of the above calculation to the second line we used a combination of the product rule and of the chain rule. To go from the second to the third line, note that because the first derivative is equal to 0 as shown immediately above of this equation, the integral in the right hand is null.

Hence, in this univariate case, the second order approximation is given by $D\left(\theta ,\theta_{0}\right)\approx \Phi (1)+\frac{1}{2}\Phi^{\prime \prime }(1){\left(\theta -\theta_{0}\right)}^{2}I\left(\theta_{0}\right)$, where $I\left(\theta_{0}\right)$ is Fisher’s information. Thus, the average discrepancy between an approximating and a generating model is scaled by the inverse of the theoretical variance of the Maximum Likelihood estimator, regardless of the form of the function Φ().

#### 2.1.3. Insight 3: Setting Φ(*t*) = −2 log *t* Connects $D\left(\theta ,\theta_{0};\Phi \right)$ With Entropy and Information Theory

Akaike proceeded to arbitrarily set the function Φ(*t*) to Φ(*t*) = −2 log *t*. Using this function not only furthered the connection with ML theory, but also introduced the connection of his thinking with Information Theory. By using this arbitrary function, the average discrepancy becomes a divergence because $D\left(\theta_{0},\theta_{0}\right)=\Phi (1)=0$ and the approximation of the average discrepancy, heretofore denoted as $W\left(\theta ,\theta_{0}\right)$, is modulated by Fisher’s information, the variance of the Maximum Likelihood estimator: $D\left(\theta ,\theta_{0}\right)\approx W\left(\theta ,\theta_{0}\right)={\left(\theta -\theta_{0}\right)}^{2}I\left(\theta_{0}\right)$. For a multivariate *θ*_0_ we get then that $W\left(\theta ,\theta_{0}\right)={\left(\theta -\theta_{0}\right)}^{\prime }I\left(\theta_{0}\right)\left(\theta -\theta_{0}\right)$ where $I\left(\theta_{0}\right)$ is Fisher’s Information matrix (Pawitan, 2001). Conveniently then, the arbitrary factor of 2 gave his general average discrepancy function the familiar “neg-entropy” or Kullback-Leibler (KL) divergence form

(2) $$\begin{array}{ll} D\left(\theta ,\theta_{0}\right) & =-2\int f\left(x;\theta_{0}\right)\text{log}\left(\frac{f(x;\theta )}{f\left(x;\theta_{0}\right)}\right)dx\\ & =-2E_{X}\left[\text{log}\frac{f(X;\theta )}{f\left(X;\theta_{0}\right)}\right]\\ & =-2\left[E_{X}(\text{log}f(X;\theta ))-E_{X}\left(\text{log}f\left(X;\theta_{0}\right)\right)\right]\\ & =2E_{X}\left(\text{log}f\left(X;\theta_{0}\right)\right)-2E_{X}(\text{log}f(X;\theta ))\\ & \;=2KL\left(\theta ,\theta_{0}\right) \end{array}$$

thus bringing together concepts in ML estimation with a wealth of results in Information Theory. The two expectations (integrals) in the last line of the above equation were often succinctly denoted by Akaike as *Sgg* and *Sgf*, respectively: these are the neg-selfentropy and the neg-crossentropy terms. Thus, he would write that last line as 2*KL*(*θ*, *θ*_0_) = 2[*Sgg* − *Sgf*]. Note that for consistency with Akaike (1973) we have retained his notation and in particular, the order of arguments in the KL function, as opposed to the notation we use in Dennis et al. (2019).

#### 2.1.4. Insight 4: $D\left(\theta ,\theta_{0}\right)$ Is Minimized at the ML Estimate of *θ*

Aikaike’s fourth critical insight was to note that a Law of Large Numbers (LLN) approximation of the Kullback-Leibler divergence between the true, generating stochastic process and a statistical model is minimized by evaluating the candidate model at its maximum likelihood estimates. Such conclusion can be arrived at even if the generating stochastic model is not known. Indeed, given a sample of size *n*, *X*_1_, *X*_2_, …, *X*_*n*_ from the generating model, from the LLN we have that

$${\hat{D}}_{n}\left(\hat{\theta },\theta_{0}\right)=-2\times \frac{1}{n}\overset{n}{\underset{i=1}{\sum }}\text{log}\frac{f\left(x_{i};\hat{\theta }\right)}{f\left(x_{i};\theta_{0}\right)},$$

which is minimized at the ML estimate $\hat{\theta }$. Akaike actually thought that this observation could be used as a *justification for the maximum likelihood principle*: “Though it has been said that the maximum likelihood principle is not based on any clearly defined optimum consideration, our present observation has made it clear that it is essentially designed to keep minimum the estimated loss function which is very naturally defined as the mean information for discrimination between the estimated and the true distributions” Akaike (1973).

#### 2.1.5. Insight 5: Minimizing $D\left(\theta ,\theta_{0}\right)$ Is an Average Approximation Problem

Akaike’s fifth insight was to recognize the need to account for the randomness in the ML estimator. Because multiple realizations of a sample *X*_1_, *X*_2_, …, *X*_*n*_ each results in different estimates of *θ*, the average discrepancy should be considered a random variable. The randomness hence, is with respect to distribution of the maximum likelihood estimator $\hat{\theta }$. Let $R\left(\theta_{0}\right)=E_{\hat{\theta }}\left[D\left(\hat{\theta },\theta_{0}\right)\right]$ denote our target average over the distribution of $\hat{\theta }$. Then, the problem of minimizing the Kullback Leibler divergence can be conceived as an approximation problem where the target is the average:

$$\begin{array}{ll} R\left(\theta_{0}\right)=E_{\hat{\theta }}D\left(\hat{\theta },\theta_{0}\right) & =2E_{\hat{\theta }}[E_{X}\left(\text{log}f\left(X;\theta_{0}\right)\right)\;-E_{X}(\text{log}f(X;\hat{\theta })|\hat{\theta })]\\ & =2E_{X}\left(\text{log}f\left(X;\theta_{0}\right)\right)\;-2E_{\hat{\theta }}\left[E_{X}(\text{log}f(X;\hat{\theta })|\hat{\theta })\right]. \end{array}$$

In the final expression of the equation above, the first term is an unknown constant. The second term on the other hand, is the expected value of a conditional expectation.

#### 2.1.6. Insight 6: $D\left(\theta ,\theta_{0}\right)$ Can Be Approximated Geometrically Using Pythagoras’ Theorem

Instead of estimating the expectations above, Akaike thought of substituting the probabilistic entropy $D\left(\hat{\theta },\theta_{0}\right)$ with its Taylor Series approximation $W\left(\hat{\theta },\theta_{0}\right)={\left(\hat{\theta }-\theta_{0}\right)}^{\prime }I\left(\theta_{0}\right)\left(\hat{\theta }-\theta_{0}\right)$, which can then be interpreted as a squared statistical distance. This approximation is indeed the square of a statistical distance wherein the divergence between any two points $\hat{\theta }$ and *θ*_0_ is weighted by their dispersion in multivariate space, measured by the eigenvalues of the positive definite matrix $I\left(\theta_{0}\right)$. This sixth insight led him straight into the path to learning about the KL divergence between a generating process and a set of proposed probabilistic mechanisms/models. By viewing this quadratic form as a statistical distance, Akaike was able to use a battery of clear measure-theoretic arguments relying on various convergence proofs to derive the AIC.

**FIGURE 1 |:**
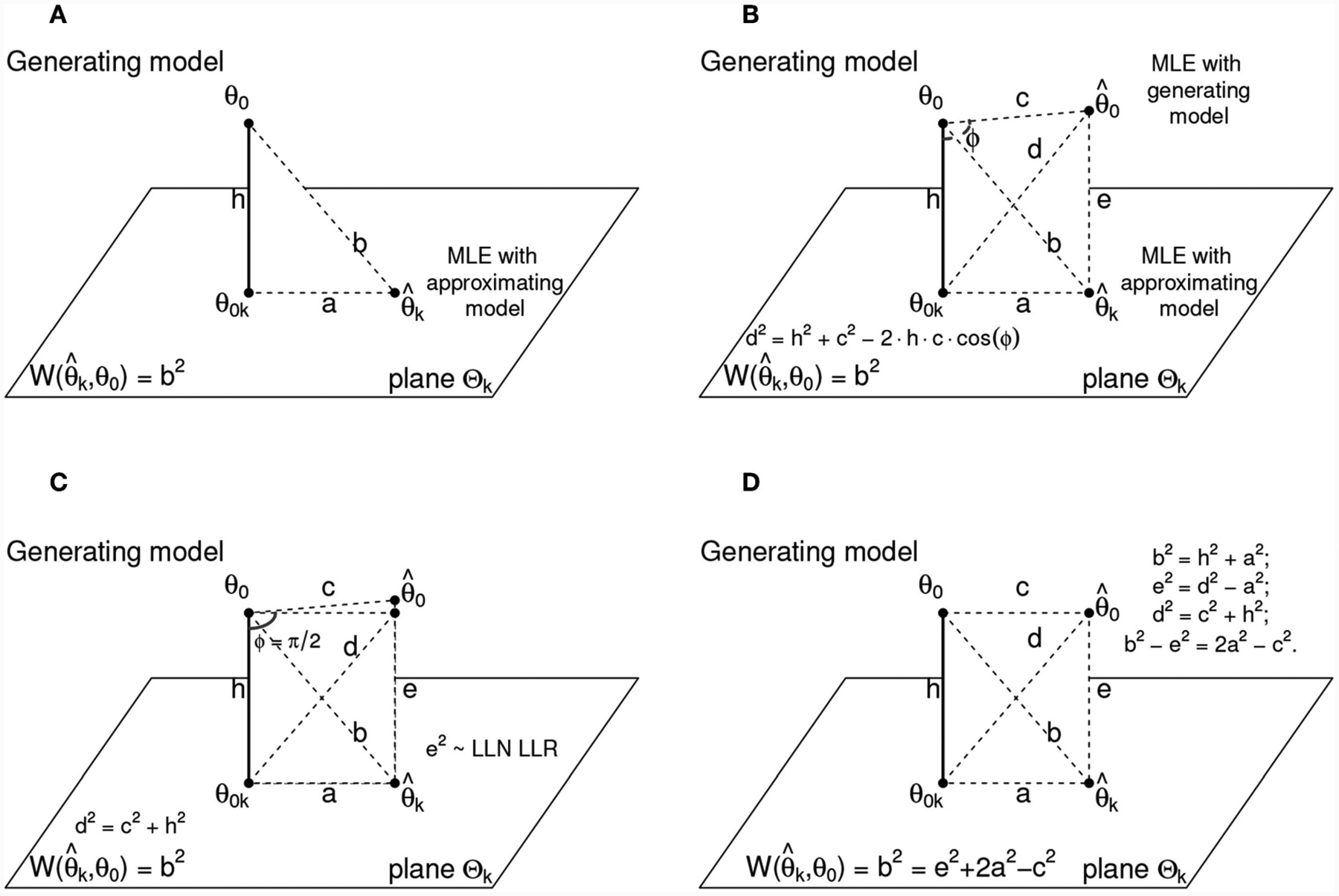
The geometry of Akaike’s Information Criterion. **(A)** Shows *θ*_0_, which is the generating model and *θ*_0*k*_ which is the orthogonal projection of the generating model into the space Θ_*k*_ of dimension *k*. ${\hat{\theta }}_{k}$ is the ML estimate (MLE) of an approximating model of dimension *k* given a data set of size *n*. Akaike’s objective was to solve for *b*^2^, which represents in this geometry $W\left(\hat{\theta },\theta_{0}\right)$, the quadratic form approximation of the divergence between the generating and the approximating models. Akaike showed that ${\hat{\theta }}_{k}$ can be thought of as the orthogonal projection of the MLE of ${\hat{\theta }}_{0}$ **(B)**. This last quantity ${\hat{\theta }}_{0}$ represents the MLE of *θ*_0_ with a finite sample of size *n* and assuming that the correct model form is known. The angle *ϕ* is not necessarily a right angle, but Akaike used *ϕ* ≈ *π*/2 so that the generalized Pythagoras theorem [equation on the lower left side of **(B)**] could be approximated with the simple version of Pythagoras [equation on the lower left side of **(C)**] when the edge *h* is not too long. When implemented, this Pythagoras equation can be used in conjunction with the other Pythagorean triangles in the geometry to solve for the squared edge *b*. The equations leading to such solution are shown in **(D)**.

Interestingly, and although he doesn’t explicitly mentions it in his paper, his entire argument can be phrased geometrically: if the average discrepancy that he was after could be approximated with the square of a statistical distance, its decomposition using Pythagoras theorem was the natural thing to do. By doing such decomposition, one can immediately visualize the ideas in his proof with a simple sketch. We present such sketch in Figure 1. In that figure, the key triangle with a right angle has as vertices the truth *θ*_0_ of unknown dimension *L*, the ML estimator $\hat{\theta }$ of dimension *k* ≤ *L*, denoted ${\hat{\theta }}_{k}$ and finally, *θ*_0*k*_. This quantity represents the orthogonal projection of the truth in the plane where all estimators of dimension *k* lie, which is in turn denoted as Θ_*k*_ (Figure 1A). Figure 1B shows a fourth crucial point in this geometrical interpretation: it is the estimator of *θ*_0_ from the data using a model with the same model form as the generating model, but with parameters estimated from the data. To distinguish it from ${\hat{\theta }}_{k}$ we denote this estimator ${\hat{\theta }}_{0}$. Because it has the same dimensions than the generating model, ${\hat{\theta }}_{0}$ can be thought of as being located in the same model surface as the generating model *θ*_0_. Akaike’s LLN approximation of the KL divergence as an average of log-likelihood ratios ${\hat{D}}_{n}\left(\hat{\theta },\theta_{0}\right)=-2\times \frac{1}{n}\sum_{i=1}^{n}\text{log}\frac{f\left(x_{i};\hat{\theta }\right)}{f\left(x_{i};\theta_{0}\right)}$ comes to play in this geometric derivation as the edge labeled *e*^2^ in Figure 1B that traces the link between ${\hat{\theta }}_{0}$ and the ML estimator ${\hat{\theta }}_{k}$. Following Akaike’s derivation then, the ML estimator ${\hat{\theta }}_{k}$ can be thought as the orthogonal projection of ${\hat{\theta }}_{0}$ onto the plane Θ_*k*_.

Before continuing with our geometric interpretation, we alert the reader that in Figure 1 all the edges are labeled with a lowercase letter with the purpose of facilitating this geometric visualization. The necessary calculations to understand Akaike’s results are presented as simplified algebraic calculations but the reader however, is warned that these edges or lower case letters denote for the most part random variables. We leave these simple letters here because in Akaike’s original derivations, the technical measure-theoretic operations may end up distracting the reader from a natural geometric understanding of the AIC.

In simple terms then, the objective of this geometric representation is to see that obtaining an estimate of the discrepancy between the approximating model and the generating process amounts to solving for the square of the edge length *b*, which is in fact the KL divergence quadratic form approximation. That is, $b^{2}=W\left(\hat{\theta },\theta_{0}\right)$. Proceeding with our geometric interpretation, note that the angle *ϕ* between edges h and c in Figure 1B is not by necessity a right angle, and that the generalized Pythagoras Theorem to find the edge length *d* applies. Akaike then noted that provided that the approximating model is in the vicinity of the generating mechanism, the third term of the generalized Pythagoras form of the squared distance *d*^2^ = *c*^2^ + *h*^2^ − 2*ch* cos *ϕ* was negligible when compared with *c*^2^ and *h*^2^ [see Akaike, 1973, his Equation (4.15) and his comment about that term in the paragraph above his Equation (4.19). See also De Leeuw 1992, text under his Equation (4)], and so he proceeded to simply use only the first two terms, *c*^2^ and *h*^2^ (see Figure 1C). The immense success of the AIC in a wide array of scientific settings to date shows that this approximation, as rough as it may seem, is in fact quite reliable. This approximation allowed him to write the squared distance *d*^2^ in two different ways: as *d*^2^ ≈ *c*^2^ + *h*^2^ and as *d*^2^ = *a*^2^ + *e*^2^. Because by construction, we have that *b*^2^ = *h*^2^ + *a*^2^, one can immediately write the difference *b*^2^ − *e*^2^ as

$$\begin{array}{ll} b^{2}-e^{2} & =h^{2}+a^{2}-d^{2}+a^{2}\\ & =h^{2}+a^{2}-c^{2}-h^{2}+a^{2}, \end{array}$$

and then solve for *b*^2^ (see Figure 1D):

(3) $$b^{2}=e^{2}+2a^{2}-c^{2}.$$

Using asymptotic expansions of these squared terms, the observed Fisher’s information and using known convergence in probability results, Akaike showed when multiplied by the sample size *n*, the difference of squares *c*^2^ −*a*^2^ was approximately chi-squared distributed with degrees of freedom *L* − *k* and that $na^{2}\sim \chi_{k}^{2}$. Then, multiplying equation (3) by *n* gives

$$nb^{2}=nW\left({\hat{\theta }}_{k},\theta_{0}\right)\approx \underset{=2\times \text{log-likelihood ratio }}{\underset{︸}{nD_{n}\left({\hat{\theta }}_{k},{\hat{\theta }}_{0}\right)}}+\underset{\sim \chi_{k}^{2}}{\underset{︸}{na^{2}}}-\underset{\sim \chi_{L-k}^{2}}{\underset{︸}{n\left(c^{2}-a^{2}\right)}}.$$

Finally, one may arrive at the original expected value of the conditional expectation shown above by replacing the chi-squares with their expected values, which are given by their degrees of freedom. Hence,

(4) $$\begin{array}{ll} nE_{{\hat{\theta }}_{k}}\left[W\left({\hat{\theta }}_{k},\theta_{0}\right)\right]\; & \approx nD_{n}\left({\hat{\theta }}_{k},{\hat{\theta }}_{0}\right)+2k-L,\text{ or}\\ E_{{\hat{\theta }}_{k}}\left[W\left({\hat{\theta }}_{k},\theta_{0}\right)\right] & \approx \frac{-2}{n}\overset{n}{\underset{i=1}{\sum }}\text{log}f\left(x_{i};{\hat{\theta }}_{k}\right)+\frac{2k}{n}-\frac{L}{n}\;+\frac{2}{n}\overset{n}{\underset{i=1}{\sum }}\text{log}f\left(x_{i};{\hat{\theta }}_{0}\right). \end{array}$$

**FIGURE 2 |:**
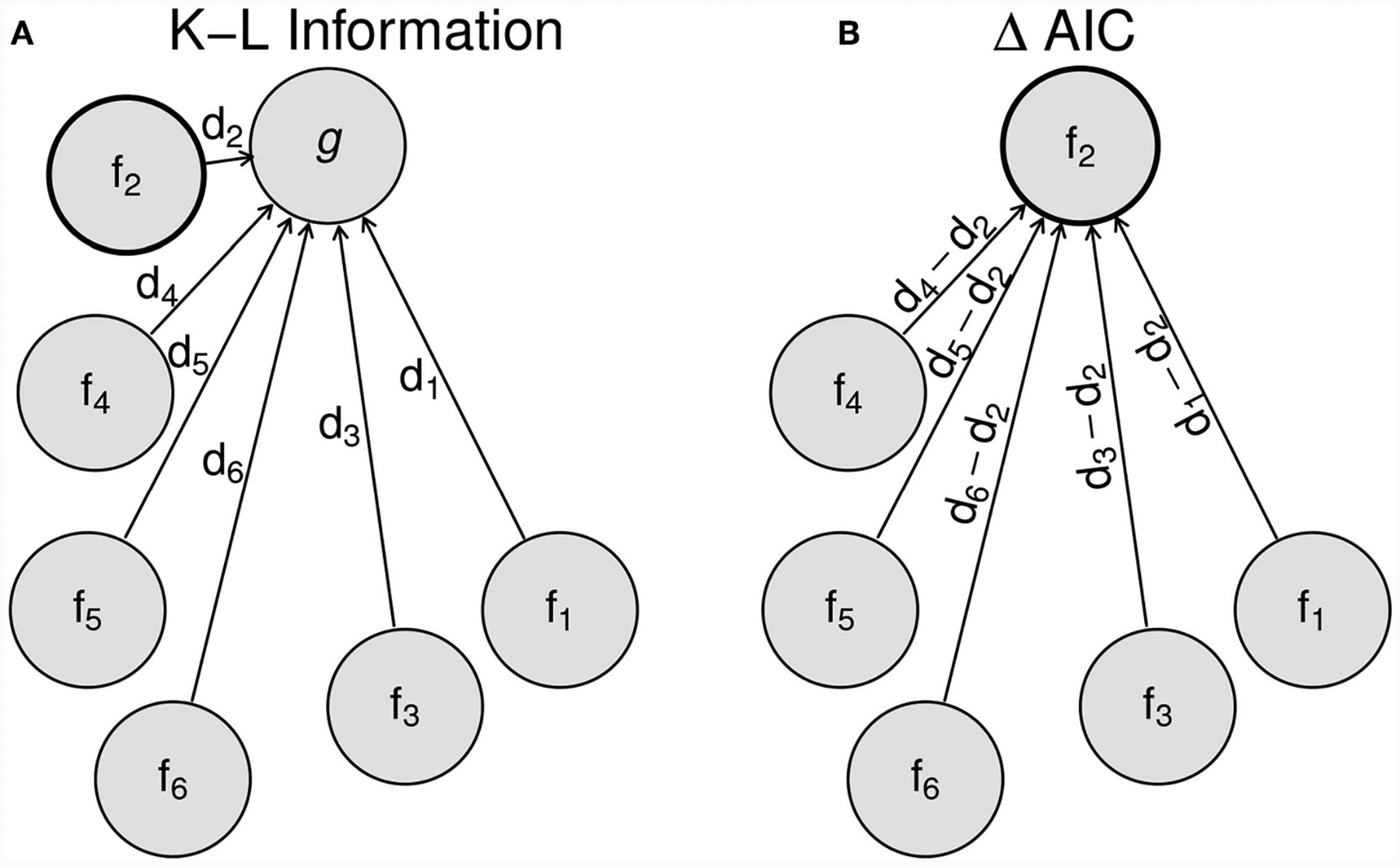
Schematic representation of the logic of multi-model selection using the AIC. *g* represents the generating model and *f*_*i*_ the *i*^*th*^ approximating model. The Kullback-Leibler information discrepancies (*d*_*i*_) are shown on the left **(A)** as the distance between approximating models and the generating model. The ΔAICs shown on the right **(B)** measures the distance from approximating models to the best approximating model. All distances are on the information scale.

The first two terms in the above expression, $-2\sum_{i=1}^{n}\text{log}f\left(x_{i};{\hat{\theta }}_{k}\right)+2k$, constitute what came to be known as the AIC. These terms correspond respectively to twice the negative log-likelihood evaluated at the MLE and twice the number of parameters estimated in the approximating model. To achieve multi-model comparison (see Figure 2), Akaike swiftly pointed out that in fact, only these first two terms are needed because the true model dimension *L* and the term $\sum_{i=1}^{n}\text{log}f\left(x_{i};{\hat{\theta }}_{0}\right)$ both terms (1) remain the same across models, as long as the same data set is used and (2) *cannot be known* because they refer to the true model dimension. Akaike rightly noted that if one were to compute Equation (4) for a suite of approximating models, these two terms would remain the same across all models and hence, could in practice be ignored for comparison purposes: these unknowns then act as constants of proportionality that are invariant to model choice. Therefore, in order to compare the value of this estimated average discrepancy across a suite of models, the user only needs to calculate the AIC score $-2\sum_{i=1}^{n}\text{log}f\left(x_{i};{\hat{\theta }}_{k}\right)+2k$ for each model and deem as best that model for which the outcome of this calculation is the smallest. The logic embedded in Akaike’s reasoning is represented graphically in Figure 2 (redrawn from Burnham et al., 2011). This reasoning kickstarted the practice, still followed in science 46 years later, to disavow the absolute truth in favor of a careful examination of multiple, if not many, models.

Finally, the reader should recall that what Equation (4) is in fact approximating is

(5) $$R\left(\theta_{0}\right)=E_{\hat{\theta }}D\left({\hat{\theta }}_{k},\theta_{0}\right)=-2E_{\hat{\theta }}\left[E_{X}\left(\text{log}f\left(X;{\hat{\theta }}_{k}\right)|{\hat{\theta }}_{k}\right)\right]\;+2E_{X}\left(\text{log}f\left(X;\theta_{0}\right)\right).$$

and that this last expression is in fact the expectation with respect to ${\hat{\theta }}_{k}$ of

(6) $$-2\int f\left(x;\theta_{0}\right)\text{log}\frac{f\left(x;{\hat{\theta }}_{k}\right)}{f\left(x;\theta_{0}\right)}dx=-2\int f\left(x;\theta_{0}\right)\text{log}f\left(x;{\hat{\theta }}_{k}\right)dx\;+2\int f\left(x;\theta_{0}\right)\text{log}f\left(x;\theta_{0}\right)dx.$$

Later, Akaike (1974) referred to the integral $\int f\left(x;\theta_{0}\right)\text{log}f\left(x;{\hat{\theta }}_{k}\right)dx$ as *Sgf* and to *∫ f* (*x*; *θ*_0_) log *f* (*x*; *θ*_0_)*dx* as *Sgg*, which are names easy to remember because it’s almost as if the *S* in *Sgf* and *Sgg* represent the integral sign and *g* and *f* are a short hand representation of the probability density function of the generating stochastic process and of the approximating model, respectively.

One of our central motivations to write this paper is the following: by essentially ignoring the remainder terms in Equation (4), since 1973 practitioners have been almost invariably selecting the “least worst” model among a set of models (but see Spanos, 2010). In other words, we as a scientific community, have largely disregarded the question of how far, *in absolute terms not relative*, is the generating process from the best approximating model. Suppose the generating model is in fact very far from all the models in a set of models currently being examined. Then, the last term in Equation (4) will be very large with respect to the first two terms for all the models in a model set that is being examined, and essentially any differences between the terms $-2\sum_{i=1}^{n}\text{log}f\left(x_{i};{\hat{\theta }}_{k}\right)+2k$ for every model will be meaningless.

### 2.2. The Problem of Multiple Models

Akaike’s realization that “truth” did not need to be known in order to select from a suite of models which one was closest to truth shaped the following four and a half decades of scientific undertaking of model-centered science. Scientists were then naturally pushed toward the confrontation of not one or two, but multiple models with their experimental and observational data. Such approach soon led to the realization that basing the totality of the inferences on the single best model was not adequate because it was often the case that a small set of models would appear indistinguishable from each other when compared (Taper and Ponciano, 2016b).

Model averaging is by far, the most common approach used today to make inferences and predictions following an evaluation of multiple models *via* the AIC. Multiple options to do model averaging exist but in all cases, this procedure is an implicit Bayesian methodology that results in a set of posterior probabilities for each model. These posterior probabilities are called the “Akaike weights.” For the *i*^*th*^ model in a set of candidate models, this weight is computed as

$$w_{i}=\frac{{\text{e}}^{\left(-\Delta_{i}/2\right)}}{\overset{R}{\underset{r=1}{\sum }}{\text{e}}^{\left(-\Delta_{r}/2\right)}}.$$

In this expression, Δ_*i*_ is the *i*^*th*^ difference between the AIC value and the best (i.e., the lowest) AIC score in the set of *R* candidate models. Although this definition is very well-known, cited and used (Taper and Ponciano, 2016b), it is seldom acknowledged that because these weights are in fact posterior probabilities, they must result from adopting a specific set of subjective model priors. Burnham et al. (2011) actually show that the weights shown above result from adopting the following subjective priors *q*_*i*_:

(7) $$q_{i}=C\cdot \text{exp}\left(\frac{1}{2}k_{i}\text{log}(n)-k_{i}\right),$$

where *C* is a normalization constant, *k*_*i*_ is the model dimension (the estimated number of parameters) of model *i* and *n* denotes the total sample size. Note that with sample sizes above 7, those weights increase with the number of parameters, thus favoring parameter rich models. The use of these priors makes model averaging a confirmation approach (Bandyopadhyay et al., 2016).

For someone using evidential statistics, adopting the model averaging practice outline above presents two important problems: first, the weights are based on prior beliefs that favor more parameter rich models and are not based on actual evidence (data). Second, and much more practically, model averaging appears to artificially favor redundancy of model specification: the more models that are developed in any given region of model space, the stronger this particular region gets weighted during the model averaging process. To counter these two problems, here we propose alternatively to estimate (1) the properties of a hyperplane containing the model set, (2) the location in such plane of the best projection of the generating process and (3) an overall general discrepancy between each of the models in the model set and the generating process or truth. We achieve these goals by using the estimated KL divergences amongst all estimated models, that is, the estimated *Sf*_*i*_*f*_*j*_ for all models *i* and *j* in the candidate set. This is information that is typically ignored. Here again, we use Akaike’s mnemonic notation where *g* denotes the generating model and *f* the approximating model. Then the so called neg-crossentropy and neg-selfentropy are written as

$$\begin{array}{lll} Sgf & =\int f\left(x;\theta_{0}\right)\text{log}f\left(x;{\hat{\theta }}_{k}\right)dx & \text{and}\\ Sgg\; & =\int f\left(x;\theta_{0}\right)\text{log}f\left(x;\theta_{0}\right)dx, & \text{respectively}. \end{array}$$

In his 1974 paper, Akaike observed that the neg-crossentropy could be estimated with

(8) $$\hat{Sgf}=\frac{1}{n}\overset{n}{\underset{i=1}{\sum }}\text{log}f\left(x_{i};{\hat{\theta }}_{k}\right)-\frac{k}{n}=-\frac{AIC}{2n}.$$

We wish to point out that in the “popular” statistical literature within the Wildlife Ecology sciences (e.g., Burnham and Anderson, 2004; Burnham et al., 2011), it is often repeated that an estimator of $E_{\hat{\theta }}\left[E_{X}\left(\text{log}f\left(X;{\hat{\theta }}_{k}\right)|{\hat{\theta }}_{k}\right)\right]$ is given by −*AIC*/2. In fact, Akaike (1974) shows that the correct estimator is given by Equation (8). This distinction, albeit subtle, marks a difference when the analyst wishes to compare not only which model best approximates the generating process, but also the strength of the evidence for one or the other model choice.

In what follows, we extend Akaike’s geometric derivation to make inferences regarding the spatial configuration of the ensemble of models being considered as approximations to the generating process. As we show with an ecological example, unlike model averaging this natural geometric extension of the AIC is fairly robust to the specification of models around the same region of model space and is actually aided, not hampered, by proposing a large set of candidate models.

### 2.3. A Geometrical Extension of Akaike’s Extension to the Principle of Maximum Likelihood

As modelers, scientists are naturally drawn to visualize a suite of candidate models as entities in a (hyper)plane. By so doing, the geometric proximities between these entities are then intuitively understood as similarities amongst models. The key questions we answer in this paper are whether it is possible to estimate the architecture of such model space, locate a suite of approximating models within such space as well as estimating the location of the projection of truth onto that plane. All of this while not having to formulate an explicit model for the generating model. The estimation of the location of the truth projection in that plane would open the door to a formulation of an overall goodness of fit measure qualifying every single one of the AIC scores computed for a set of candidate models. Additionally, answering these questions automatically provides valuable insights to intuitively understand why or why not model averaging may be an appropriate course of action. As we show below, these questions are answerable precisely because any given set of models has a set of relationships which are typically ignored but that can be translated directly to a set of geometrical relationships that carry all the needed information and evidence.

One of the key observations of this contribution is the fact that while at the time of Akaike’s publication his approach could not be extended due to mathematical intractabilities, nowadays computer intensive methods allow the design of a straightforward algorithm to solve the model projection problem outlined above. These computational tools basically involve two methodologies: first, a numerical estimation of Kullback-Leibler (KL) divergences between arbitrary distributions and second, parallel processing to carry a Non-Metric Multidimensional (NMDS) space scaling algorithm. With the help of a NMDS, a matrix of amongst-candidate models estimated KL divergences can be transformed into an approximated Euclidean representation of models in a (hyper)plane. The coordinates of each model in that plane, that we heretofore denote (*y*_1_, *y*_2_, …) are used to solve the model projection problem. The algorithm presented here is not necessarily restricted to a two-dimensional representation of model space, but for the sake of visualization we present our development in $R^{2}$.

**FIGURE 3 |:**
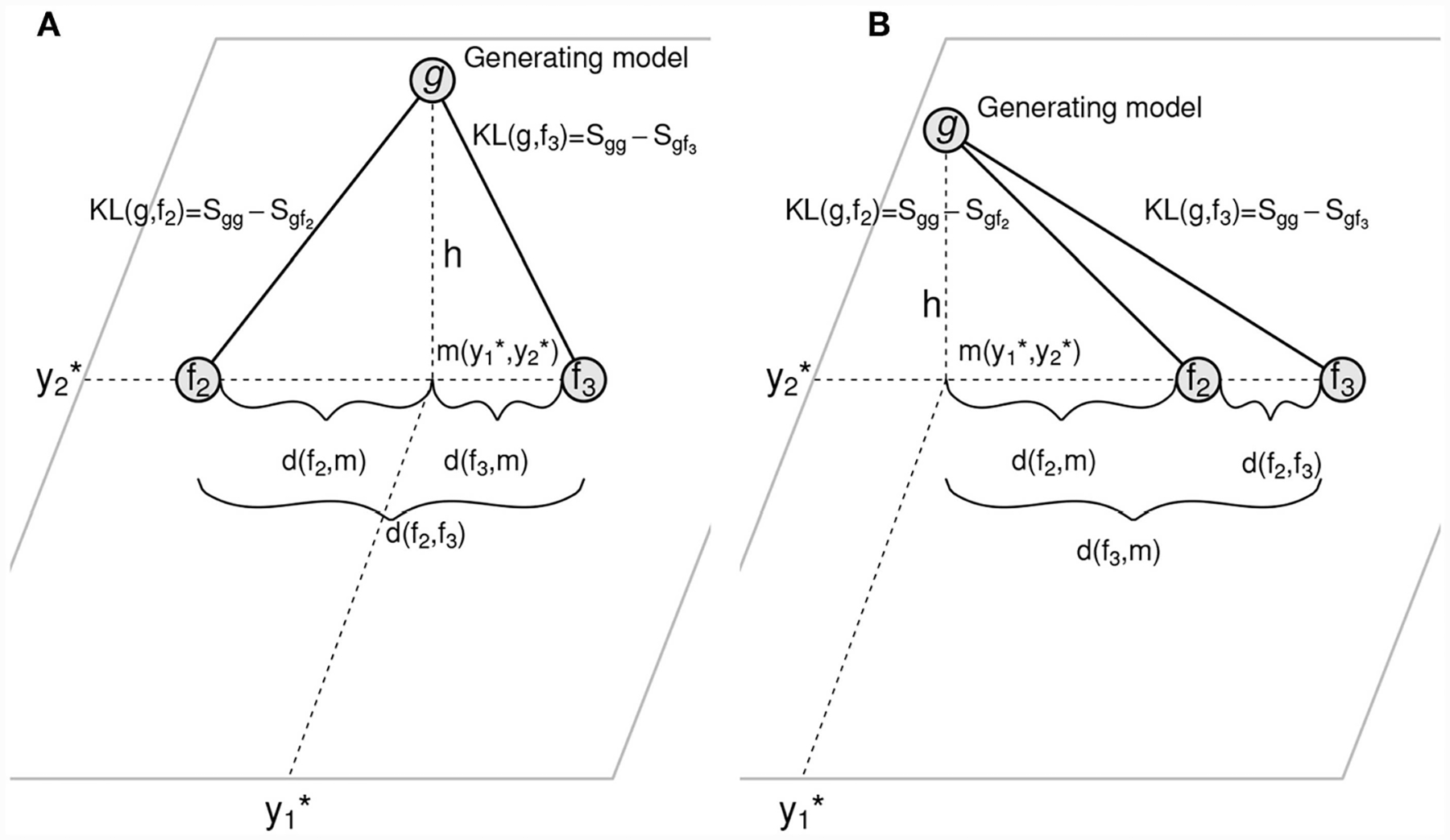
The geometry of model space. In this figure, *f*_2_ and *f*_3_ are approximating models residing in a (hyper)plane. g is the generating model. m is the projection of g onto the (hyper)plane. *d*(;)^·^ are distances between models in the plane. *d*(*f*_2_, *f*_3_) ≈ *KL*(*f*_2_, *f*_3_) with deviations due to the dimension reduction in NMDS and non-Euclidian behavior of KL divergences. As KL divergences decrease, they become increasingly Euclidian. **(A)** Shows a projection when m is within the convex hull of the approximating models, and **(B)** shows a projection when m is outside of the convex hull. Prasanta S. Bandyopadhyay, Gordon Brittan Jr., Mark L. Taper, Belief, Evidence, and Uncertainty. Problems of Epistemic Inference, published 2016 Springer International Publisher, reproduced with permission of Springer Nature Customer Service Center.

Consider the sketch in Figure 3. There, to begin with we have drawn only two approximating models *f*_2_ and *f*_3_ on a Euclidean space, along with a depiction of the location of the generating process *g* outside that plane. Such representation immediately leads to the definition of a point *m* in that plane that correspond to the orthogonal projection of the generating process onto the plane. The location of such point is denoted as $\left(y_{1}^{⋆},y_{2}^{⋆}\right)$. The length *h* in that sketch represents the deviation of the generating process from the plane of approximating models as a line from *g* to the plane that crosses such plane perpendicularly. Note also that every one of the approximating models *f*_*i*_ in that plane is situated at a distance *d*(*f*_*i*_, *m*) from the orthogonal projection *m*. In reality, both the edges as well as the points in this plane are random variables associated with a sampling error. But we ask the reader’s indulgence for the sake of the argument, just as we did above when we explained Akaike’s results, and think of these simply as points and fixed lengths. Doing so, one may also indulge, as Akaike did, in using the right-angle, simple version of the Pythagoras theorem, and assume that all the amongst-models KL divergences have a corresponding squared Euclidean distance in that representation. Then, the following equations hold

$$\{\begin{array}{lll} KL\left(g,f_{1}\right) & = & d{\left(f_{1},m\right)}^{2}+h_{1}^{2}\\ KL\left(g,f_{2}\right) & = & d{\left(f_{2},m\right)}^{2}+h_{2}^{2}\\ & \vdots & \end{array}$$

where necessarily *h*_1_ = *h*_2_ = *h*_*i*_ = … = *h*. Recalling Equation (8) we note that every one of the divergences between the approximating models and *g* can be expressed as a sum of an estimable term and a fixed, unknown term. These terms are *Sgf*_*i*_ and *Sgg*, respectively. Writing such decomposition of the KL divergences for all the equations above, and explicitly incorporating the coordinates of *m* then results in this system of equations

(9) $$\{\begin{array}{ccc} Sgg-\hat{Sgf_{1}}-d{\left(f_{1},m\left(y_{1}^{⋆},y_{2}^{⋆}\right)\right)}^{2} & = & h_{1}^{2},\\ Sgg-\hat{Sgf_{2}}-d{\left(f_{2},m\left(y_{1}^{⋆},y_{2}^{⋆}\right)\right)}^{2} & = & h_{2}^{2},\\ \vdots & \vdots & \vdots \end{array}$$

which can be solved and optimized computationally by constructing an objective function that, for any given set of values of *sgg*, $y_{1}^{⋆}$, $y_{2}^{⋆}$ in the left hand of these equations returns the sum of squared differences between all the *h*_*i*_. Because by necessity (see Figure 3) $h^{2}=h_{i}^{2}$ for all *i*, a routine minimization of this sum of squared differences can be used as the target to obtain optimal values of the unknown quantities of interest and obtain the model-projection representation shown in Figure 5. Although previously unrecognized by Taper and Ponciano (2016a), in these equations the terms *Sgg* and *h*^2^ appear always as a difference, and hence are not separable. Fortunately, a non-parametric, multivariate estimate of *Sgg* can be readily computed. We use the estimator proposed by Berrett et al. (2019), a multivariate extension of the well-known univariate estimator by Kozachenko and Leonenko (1987). Other non-parametric entropy estimators could be used if they prove to be more appropriate. For instance, the Berrett et al. (2019) estimator assumes that the data are iid. This restricts the class of problems for which we are able to separate *Sgg* and *h*^2^. An estimator for *Sgg* for dependent data would expand the class.

## 3. EXAMPLES

In what follows we illustrate our ideas and methodology with two ecological examples. The first example is an animal behavior study aiming to understand the mechanism shaping patterns of animal aggregations. The second one is an ecosystems ecology example, where the aim was to try to understand the biotic and abiotic factors that shape the species diversity and composition of a shrubland ecosystem in California.

### 3.1. An Application in Animal Behavior

The phenomenon of animal aggregations has long been the focus of interest for evolutionary biologists studying behavior (Brockmann, 1990). In some animal species, males form groups surrounding females, seeking breeding opportunities. Often, these mating groups vary substantially in size, even during the same breeding season and breeding occasion. This is particularly true in some species with external fertilization where females spawn the eggs and one or more males may fertilize them. The females of the American horseshoe crab, *Limulus polyphemus* leave sea “en masse” to spawn at the beach during high tide, 1–4 times a year. As females enter the beach and find a place to spawn, males land in groups and begin to surround the females. Nesting typically occur in pairs, but some females attract additional males, called satellites, and spawn in groups. As a result, when surveys of the mating group size are done, one may encounter horseshoe crab pairs with 0, 1, 2, 3, … satellite males. That variation in the number of satellite males is at the root of the difficulty in characterizing the exact make-up of the crab population. Hence, for years during spawning events, Brockmann (1990) focused on recording not only the total number of spawning females in a beach in Seahorse Key (an island along Florida’s northern west coast) but also the number of satellite males surrounding each encountered pair. Those data have long been the focus of attempts at a probabilistic description of the distribution of the number of satellite males surrounding a pair of horseshoe crabs using standard distribution models (e.g., Poisson, zero inflated Poisson, negative binomial, zero inflated negative binomial, hurdle-negative binomial distributions).

When one of us (JMP) met H. J. Brockmann in 2010, she asked the following: “how will fitting different discrete probability distributions to my data help me understand the biological mechanisms underlying group formation in this species?” After years of occasional one-on one meetings and back and forth discussions, we put together a detailed study (Brockmann et al., 2018) in which we compared the observed distribution of the number of satellites surrounding a female to the same distribution resulting from a complex, individual-based model simulation program. Importantly, this individual-based model allowed us to translate different hypotheses regarding the influence of different factors, like female density or male density around a female, into the decision by a new satellite male of joining a mating group or continuing the search.

The comparison between the real data and the simulated data *via* discrete probability distributions then allowed these authors to identify the biological settings that resulted in *in silico* distributions of satellites that most resembled the real, observed distributions of satellite males. To do that comparison, Brockmann et al. (2018) first fitted a handful of discrete probability models to the counts of the number of satellites surrounding each pair from each one of *N* = 339 tides, and proceeded to find the standard probability model that best described the data. These authors then fitted the same models to the simulated data sets under different biological scenarios and found the simulation setting that yielded the highest resemblance between the real data and the digital data. Finally Brockmann et al. (2018) discuss the implications of the results.

One of the most relevant conclusions of these authors was that their comparative approach was useful as a hypothesis generator. Indeed, by finding via trial and error which biological processes gave rise in the individual-based simulations to distributions of satellites that most resembled the real distributions, the researchers basically came up with a system to elicit viable biological explanations for the mechanisms shaping the distribution of the number of satellite males surrounding a pair. This approach was an attempt to answer Brockmann’s initial question to JMP.

Here, we used the simulation setting of Brockmann et al. (2018) to exemplify how our Model Projections in Model Space (MPMS) approach can further our understanding of what are the model attributes that make a model a good model to better understand the underlying mechanisms generating the data. By having a complex simulation program, we can describe exactly the probability distribution of the data-generating process and we can validate our MPMS approach.

In what follows we first explain how we fitted our proposed models to the tides’ count data, and then how we compute the quantities needed to generate an approximate representation of models in model space that includes the estimated projection of the true, data-generating process.

#### 3.1.1. Likelihood Function for the Satellites Count Data

A handful of discrete probability models can be fit conveniently to the male satellites counts data using the same general likelihood functions by means of a reduced-parameter multinomial distribution model parameterization. As we will see below, this reduced-parameter multinomial likelihood formulation is instrumental to compute analytically the KL divergences between each one of the models as well as the neg-selfentropy. Many modern biological models, like phylogenetic Markov models, use this reduced-parameter formulation (Yang, 2000), and the example presented here can be readily used in many other settings in ecology and evolution (e.g., Rice, 1995).

In this example we adopt the following notation: the probability mass function of each discrete probability model *i* (*i* = 1, 2, … *r* where *r* is the number of models in the model set) is denoted as *f*_*i*_(*x*). Following Brockmann et al. (2018), we use *f*_1_(*x*) to denote the Poisson distribution (Poisson), *f*_2_(*x*) the negative binomial distribution (NegBin), *f*_3_(*x*) the zero inflated Poisson distribution (ZIP), *f*_4_(*x*) the zero inflated negative binomial distribution (ZINegBi), *f*_5_(*x*) a hurdle negative binomial distribution (HurdNBi), *f*_6_(*x*) a Poisson-negative binomial mixture (PoiNB), *f*_7_(*x*) a negative-binomial-Poisson mixture (NBPois), *f*_8_(*x*) a one-inflated Poisson distribution (OIPoiss), and *f*_9_(*x*) a one inflated negative-binomial distribution (OINegBi). In this example, *r* = 9.

We begin with the likelihood function for the counts for one tide, and extend it to the ensemble of counts for *N* tides Because for each tide *j*, *j* = 1, 2, …, *N* the data consisted of the number of 0’s, 1’s, etc…, the data can be represented as a multinomial sample with *k* categories and probabilities *π*_1_, *π*_2_, …, *π*_*k*_: Let *Y*_1_ be the number of pairs with no satellites found at the beach in one tide, *Y*_2_ the number of pairs with 1 satellite male in one tide, *Y*_3_ the number of pairs with 2 satellite males in one tide, …, *Y*_*k*−1_ the number of pairs with *k* − 2 satellites in one tide and *Y*_*k*_ the number of pairs with *k* − 1 or more satellites in one tide. Suppose for instance that we are to fit the Poisson distribution model with parameter λ to the counts of one tide. Then, the reduced parameter multinomial distribution arranged to fit the Poisson model would be parameterized using the following probabilities for each category:

(10) $$\begin{array}{llll} \pi_{1} & =P(X=0) & =f_{1}(0) & =e^{-\lambda },\\ \pi_{2} & =P(X=1) & =f_{1}(1) & =\lambda e^{-\lambda },\\ \pi_{3} & =P(X=2) & =f_{1}(2) & =\frac{\lambda^{2}e^{-\lambda }}{2!},\\ & \vdots & & \\ \pi_{k-1} & =P(X=k-2) & =f_{1}(k-2) & =\frac{\lambda^{k-2}e^{-\lambda }}{(k-2)!}\\ \pi_{k} & =P(X\geq k-1) & =1-\sum_{s=0}^{k-2}f_{1}(s) & =1-\sum_{s=0}^{k-2}\frac{\lambda^{s}e^{-\lambda }}{(s)!}. \end{array}$$

It follows that if in a given tide *j* a total of *n*_*j*_ pairs are counted and *y*_*j*,1_ is the number of females with no satellites, *y*_*j*,2_ is the number with one satellites, etc., such that $\sum_{i=1}^{k}y_{j,k}=n_{j}$, the likelihood function needed to fit the Poisson probability model to the data of one tide is simply written as:

$$\begin{array}{ll} L_{j}(\lambda ) & =P\left(Y_{j,1}=y_{j,1},Y_{j,2}=y_{j,2},\ldots ,Y_{j,k-1}=y_{j,k-1},Y_{j,k}=y_{j,k}\right)\\ & =\frac{n!}{y_{j,1}!y_{j,2}!y_{j,3}!\ldots y_{j,k}!}\pi_{1}^{y_{j,1}}\pi_{1}^{y_{j,2}}\ldots \pi_{k}^{y_{j,k}}, \end{array}$$

and the overall likelihood function for the *N* tides is simply

$$L(\lambda )=\overset{N}{\underset{i=1}{\prod }}L_{j}(\lambda ).$$

Finally, note that for this reduced parameter multinomial model, the ML expected frequencies would simply be computed as $n_{j}{\hat{\pi }}_{1}$. For example, under the Poisson model, the expected number of 0’s in a sample would be computed as $n_{j}{\hat{\pi }}_{1}=P\hat{\left(X=0\right)}=e^{-\hat{\lambda }}$, where $\hat{\lambda }$ denotes the ML estimate of λ.

The likelihood function and each of the predicted probabilities for every model were computed using the programs in the files CrabsExampleTools.R and AbundanceToolkit2.0.R downloadable from our github webpage, which works as follows. Suppose that for a single tide, the counts of the number of pairs with 0, 1, 2, 3, 4, and 5 or more satellites are 112, 96, 101, 48, 22, 16, respectively. Then, the program abund.fit (found in the set of functions AbundanceToolkit2.0.R) takes those counts and returns, for every model in a pre-specified model set, the expected frequencies (from which the probabilities of every category in the reduced-parameter multinomial are retrievable), the ML estimates of each set of model parameters, the maximized log-likelihood and other statistics.

The processes of simulating any given number of tide counts according to Brockmann et al. (2018) and computing the ML estimates and other statistics for every model and every tide in a pre-specified model set are packaged within our function short.sim() whose output is (1) a matrix of simulated counts, with one row per tide. In each row the data for a single tide is displayed from left to right, showing the number of pairs with 0, 1, 2, 3, 4, and 5 or more satellites. (2) a list with the statistics (ML estimates, maximized log-likelihood, predicted counts, etc…) for every model and every tide. (3) A matrix of information criteria values for every tide (row) and every model (column) in the set of tested models.

#### 3.1.2. Calculation of Quantities Needed to Generate a MPMS

The generation of the MPMS necessitates solving the system of Equation (9). To solve that system of equations for any given dat set we need

**FIGURE 4 |:**
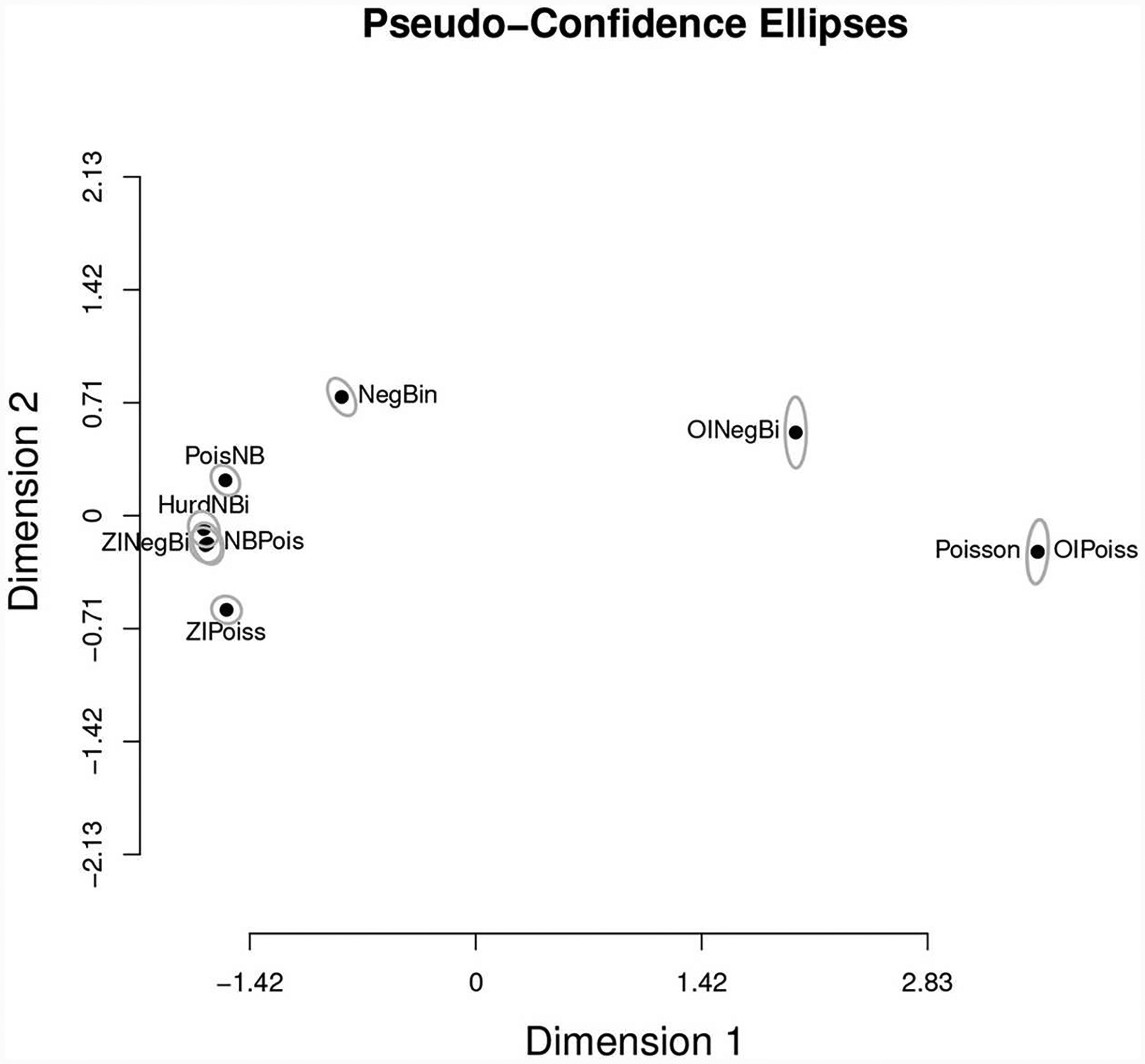
Count models for the horseshoe crab example (section 3.1) in NMDS space, along with pseudo-confidence ellipses (95%). These ellipses are based on Stress derivatives (Mair et al., 2019) and indicate in this case that the NMDS is quite stable (overall stress is 0.029).

1. A non-parametric estimate of the neg-selfentropy *Sgg*, $\hat{Sgg}$. Berrett et al. (2019) recently proposed such an estimator. Their estimator is in essence a weighted (Kozachenko and Leonenko, 1987) estimator, and uses *k* nearest neighbors of each observation as follows:
  $$H_{n}^{w}=\frac{1}{n}\overset{n}{\underset{i=1}{\sum }}\overset{k}{\underset{j=1}{\sum }}w_{j}\text{log}\xi_{(j),i},$$
  where *ξ*_(*j*),*i*_ = (*n*− 1)*e*^−*ψ*(*j*)^ *V*_*q*_∥*X*_(*j*),*i*_−*X*_*i*_∥^*q*^ with *X*_(*j*),*i*_ indicating the *j*-th nearest neighbor from the *i*-th observation *X*_*i*_. Also, in these equation *n* indicates the number of observations, *ψ*(*j*) is the digamma function and *V*_*q*_ = *π*^*q*/2^/Γ(1 + *q*/2) is the volume of the unit *q*-dimensional ball and *q* is the dimension of the multivariate observations.
  The focus of Berrett et al. (2019) was writing a complete theoretical proof of the statistical properties of their estimator. Practical guidance as to how to find these weights is however lacking in their paper, but through personal communication with T. Berrett we learned that their weights *w*_*j*_ must only satisfy the constraints (see their Equation 2):
  $$\begin{array}{ll} \sum_{j=1}^{k}w_{j} & =1\text{ and }\sum_{j=1}^{k}w_{j}\Gamma (j+2l/q)/\Gamma (j)=0\text{ for}\\ l & =1,\ldots ,q/4, \end{array}$$
  where *k* is the number of observations that define a local neighborhood of observations around any given observation. Berrett (personal communication) recommends arbitrarily choosing *k* as the sample size to the power of a third. The other restrictions on Berrett et al. (2019)’ theorem about the support of these weights were needed only for technical convenience for the proof. Berrett et al. (2019) also mentioned that for small sample sizes, the unweighted estimator may be preferable. For larger problems he recommended solving the above restrictions with a non-linear optimizator. We wrote such non-linear optimization routine to compute the weights *w*_*j*_’s and tested it extensively via simulations and embedded it into a function whose only argument is the data itself. Through extensive simulations we have verified that this routine works well for dimensions at least up to *q* = 15. We coded our optimization in R and is now part of a package of functions accompanying this paper. The function is found in the file MPcalctools.R and was named Hse.wKL. Finally, note that a typical data set for our crabs example is of dimension 6, so our routine is more than enough for a typical set of counts similar to the ones in this example. For instance, one set of counts of pairs with 0 satellites, 1 satellite, 2 satellites, …, 5 or more satellite males for one tide is *y*_1_ = 112, *y*_2_ = 96, *y*_3_ = 101, *y*_4_ = 48, *y*_5_ = 22, *y*_6_ = 16.
2. A matrix of KL divergences between all models estimated in the model set being considered. If a total of *r* models are being considered, then the elements of this matrix are {*KL*(*f*_*i*_, *f*_*j*_)}_*i*,*j*_, *i*, *j* = 1, 2, …, *r*. Computing these divergences may seem like a daunting task, especially because these quantities are, in fact, different expectations (i.e., infinite sums) evaluated at the ML estimates for each model in the model set. However, those calculations are enormously simplified by adopting the general reduced-parameter likelihood approach because the neg-crossentropy *H*(*f*_*r*_, *f*_*s*_) between two multinomial models *f*_*r*_ and *f*_*s*_ with a total sample size *n* can be computed exactly:
  (11) $$\begin{array}{ll} H\left(f_{r},f_{s}\right) & =\sum_{\left(y_{1},y_{2},\ldots ,y_{k}\right)\geq 0,\left(\sum_{k}y_{k}\right)=n}\frac{n!}{y_{1}!\ldots y_{k}!}\pi_{1,r}^{y_{1}}\ldots \;\pi_{k,r}^{y_{k}}\text{log}\left[\frac{n!}{y_{1}!\ldots y_{k}!}\pi_{1,s}^{y_{1}}\ldots \pi_{k,s}^{y_{k}}\right]\\ & =\text{log}n!+n\sum i=1^{k}\pi_{i,r}\text{log}\pi_{i,s}\;-\sum_{i=1}^{k}\sum_{y_{i=0}}^{n}\left(\begin{array}{l} n\\ y_{i} \end{array}\right)\pi_{i,r}^{y_{i}}{\left(1-\pi_{i,r}\right)}^{\left(n-y_{i}\right)}\text{log}{\text{y}}_{i}!. \end{array}$$
  Note that when *s* = *r*, then *H*(*f*_*r*_, *f*_*s*_) becomes the neg-selfentropy. Because the KL divergence is the sum of a neg-selfentropy and a crossentropy, in practice, to compute the KL divergence between two count models for a single vector of counts for one tide we only needed to compute the probabilities in Equation (10) for every model using the ML estimates for each data set and use Equation (11) above. The function in R used to compute either the neg-crossentropies or the neg-selfentropies is named H.multinom.loop() and found in the file MPcalctools.R. Following simple rules of expected values, the overall KL divergence between two count models for a set of *N* vectors of tide counts, each drawn from the same true generating process (the individual-based model simulator program), was just computed as the sum of the divergences between the two models for each vector of counts. Note that the same simplification in Equation (11) applies to the computation of the neg-selfentropy for a multinomial distribution, a fact that we used to compute the true *Sgg* for our simulator algorithm, given that the individual-based model simulator of Brockmann et al. (2018) could be used to the estimate numerically true probabilities for 0,1,2,… satellites.
3. The estimates of the neg-crossentropies $\hat{Sgf_{i}}$ and of $\hat{Sf_{i}g}$ for *i* = 1, 2, …, *r*. Although the first set of divergences, the $\hat{Sgf_{i}}$, can be estimated either using the AIC and Equation (8), by definition of the KL divergence, the estimates $\hat{Sf_{i}g}$ are in general not equal to the estimates $\hat{Sgf_{i}}$ and cannot be computed using the AIC and Equation (8). If however, *h*^2^ is very small, then using the approximation $\hat{Sgf_{i}}\approx \hat{Sf_{i}g}$ works quite well as we show in example 3.2 and in Taper and Ponciano (2016a). Fortunately, using this approximation is not always necessary and does not have to be used for a large class of statistical problems. Indeed, for the example at hand where we are fitting multiple count models and for any other case where the likelihood function may be written by means of a reduced-parameter multinomial model (like the likelihood function for most phylogenetics models, for instance), both the $\hat{Sgf_{i}}$ and the $\hat{Sf_{i}g}$ can be computed using Equation (11) by using the ML estimates of the multinomial *π*’s for each model and the ML estimates of the *π*’s for the fully parameterized (i.e., the empirical model) in lieu of the *π* parameter values for *g*. We will denote these empirical estimates (i.e., the sample proportions) as ${\bar{\pi }}_{i}$. These estimates and Equation (11) can be used to compute $\hat{Sgg}$. For a set of models and a data set including one or more tides, the estimates $\hat{Sgf_{i}}$, $\hat{Sf_{i}g}$, $\hat{Sf_{i}f_{j}}$ and $\hat{Sgg}$ are computed using the function entropies.matcalc() found in the file MPcalctools.R.
  For a simulated example where the data consisted of counts for 300 tides for which the first 5 tides were
> simdat [1:5,]012345[1,]11296101482216[2,]135125108441912[3,]14110891552316[4,]11911799601810[5,]139120117372611
The estimated matrix of neg-crossentropies for these *N* = 300 tides was
$Sfifjs.hatPoissonNegBinZIPoissZINegBiHurdNBiPoisNBNBPoisOIPoissOINegBiPoisson−4693.860−6788.198−7144.127−7261.358−7276.347−7240.670−7268.412−4694.360−5474.936NegBin−7140.595−4801.609−5748.393−5402.616−5393.795−5141.644−5417.371−7142.133−6271.978ZIPoiss−7269.760−5688.618−4778.731−4958.789−4991.042−5157.351−4973.420−7271.568−6703.770ZINegBi−7483.025−5374.228−4980.700−4792.072−4868.308−5004.579−4952.840−7484.694−6719.950HurdNBi−7504.330−5366.989−5015.873−4870.053−4792.890−4980.233−4974.241−7503.976−6688.178PoisNB−7476.723−5131.165−5178.562−5008.269−4982.275−4794.920−4975.992−7477.044−6595.687NBPois−7453.540−5389.715−4984.999−4949.951−4969.751−4970.038−4790.289−7455.437−6702.133OIPoiss−4694.328−6789.643−7145.910−7262.925−7275.909−7240.883−7270.251−4693.826−5474.358OINegBi−5606.894−6051.992−6648.368−6590.061−6559.321−6453.790−6596.554−5606.393−4735.259
The true generating process neg-selfentropy, *Sgg* was −16.01199 and the estimated neg-selfentropy $\hat{Sgg}$ was −15.96137. The real neg-crossentropies between the generating process and each of the models *Sgf*_*i*_’s and *Sf*_*i*_*g*’s were:
$SgfisPoissonNegBinZIPoissZINegBiHurdNBiPoisNBNBPoisOIPoissOINegBi−7601.606−5603.842−5485.607−5432.819−5457.657−5450.109−5463.949−7606.407−6832.673
$SfisgPoissonNegBinZIPoissZINegBiHurdNBiPoisNBNBPoisOIPoissOINegBi−7276.506−5615.999−5431.557−5414.428−5439.071−5436.292−5435.686−7281.250−6653.690
whereas the estimated neg-crossentropies were
$Sgfis.hatPoissonNegBinZIPoissZINegBiHurdNBiPoisNBNBPoisOIPoissOINegBi−7891.890−5652.049−5403.773−5200.907−5179.331−5323.294−5253.094−7891.705−7051.753
$Sfisg.hatPoissonNegBinZIPoissZINegBiHurdNBiPoisNBNBPoisOIPoissOINegBi−7638.034−5682.275−5396.418−5213.190−5193.804−5339.449−5264.447−7637.716−6906.558
4. The coordinates of every model in an NMDS space. Multidimensional scaling, MDS, is an established method (Borg et al., 2018) for representing the information in the *s* × *s* matrix *D* of distances/divergences among *s* objects as a set of coordinates for the objects in a *k*-dimensional euclidian space (*k* ≤ *s*). If *k* < *s*, there may be some loss of information. MDS has two major varieties, metric multidimensional scaling, MMDS, in which *D* is assumed to be comprised of Euclidean distances, and non-metric multidimensional scaling, NMDS, in which *D* can be made up of divergences only monotonically related to distances. The MMDS projection can be made analytically, while the NMDS projection can only be found algorithmically by iteratively adjusting the configuration to minimize a statistic known as “Stress,” which is a weighted average squared deviation of the distances between points (models in our case) calculated from the proposed configuration and the distances given in *D*.
  The matrix *D* required by NMDS should be symmetric. KL divergences are not, however, symmetric. The KL divergence can be reasonably symmetrized in a number of ways (Seghouane and Amari, 2007). We symmetrize using the arithmetic average of *KL*(*θ*_*i*_, *θ*_*j*_) and *KL*(*θ*_*j*_, *θ*_*i*_). As mentioned above in this problem we can directly calculate the symmetric KL. For other applications the symmetric *KL* can be estimated (up to the constant *Sgg*) using the KIC and its small sample version the KiCc (Cavanaugh, 1999, 2004). We follow Akaike in considering the KL divergence as a squared distance, and thus construct the matrix D from the square roots of the symmetrized KL divergence. We use the function smacofSym (De Leeuw and Mair, 2009) from the R package smacof (version 2.0, Mair et al., 2019) to calculate the NMDS. For the purposes of this paper we chose *k* = 2 so that we could have a graphical representation after augmenting the dimension to 3 to show the orthogonal distance from the generating process to its orthogonal projection *M* in the estimated plane of models. Nevertheless, the Stress of 0.029 indicates an excellent fit. Except for the very important aspect of visualization, dimension reduction is not an essential aspect of our method. Finally, the tight pseudo-confidence ellipses (95%) illustrated in Figure 4, based on Stress derivatives (Mair et al., 2019) indicate that this NMDS is quite stable.

**FIGURE 5 |:**
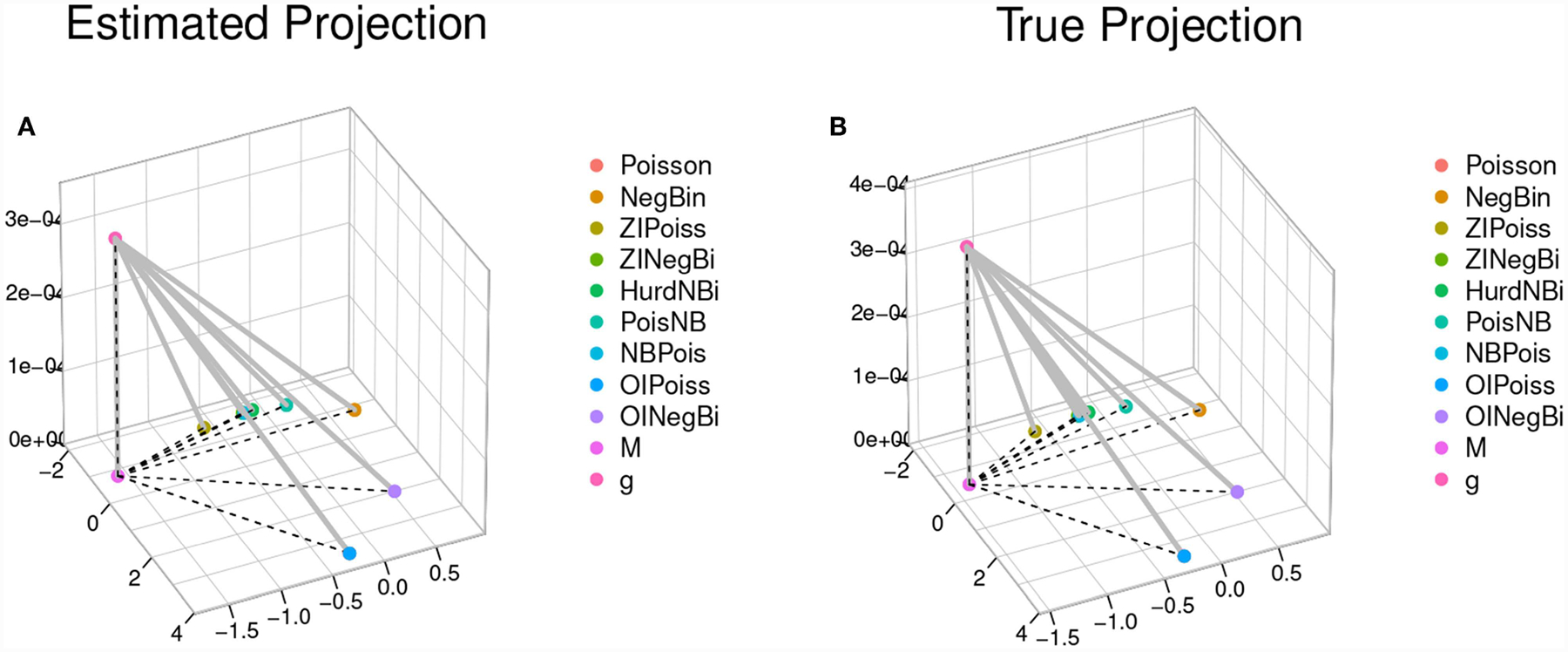
The models of Figure 2 visualized by our new methodology, and applied to our Horseshoe crab example (section 3.1). As before, *g* is the generating model and models *f*_1_, …, *f*_9_, are the approximating models and named in the legend of each panel. **(A)** Shows the estimated model projection “M” and the estimated location of the true generating process whereas **(B)** shows the location of the true model projection “M” and of the true generating process. The dashed lines are KL distances between approximating models, which were calculated according to Equation 2. The solid gray lines are the KL distances from approximating models to the generating model. The vertical dotted line shows h, the discrepancy between the generating model and its best approximation in the NMDS plane, whereas all the other dotted lines mark the discrepancy between the approximating models and the model projection “M.” A 2-dimensional representation of only the plane of models, the estimated *g* model projection and the true model projection of *g* onto that plane is shown in Figure 6.

Once all these components are computed, the system of Equation (9) can be solved with non-linear optimization. We coded such solution in the R function MP.coords found in the file *MPcalctools*.*R*. This function takes as input the estimated neg-crossentropies between all models, an estimate of *Sgg* or the neg-selfentropy of the generating process, and the vectors of estimated neg-crossentropies $\hat{Sgf_{i}}$ and $\hat{Sf_{i}g}$ to output the matrix of dimension (*r* + 1) × (*r* + 1) of symmetrized KL divergences, and the results of the NMDS with the coordinates of every model in a two-dimensional space, the estimated location of the orthogonal projection of *g* in such plane, *M*, and the estimate of *h*. Notably, this function works for any example for which these estimated quantities are available. Its output is taken by our function plot.MP to produce the three-dimensional representation of the Model Projection in Model Space shown in Figure 5. For this example, the estimated distances in the model projection space between all models, *g* and its projection *M* were
PoissonNegBinZIPoissZINegBiHurdNBiPoisNBNBPoisOIPoissOINegBiMNegBin4.46711ZIPoiss5.093581.51616ZINegBi5.214221.260690.42784HurdNBi5.224441.196990.523470.09773PoisNB5.108580.895610.813090.425420.33690NBPois5.198831.243480.432080.017980.091390.41228OIPoiss0.001814.468595.095305.215885.226095.110185.20050OINegBi1.695002.853323.738753.768183.759263.587463.751251.69618M4.512352.332801.265661.674511.761401.974441.673874.514163.50306g4.512352.332801.265661.674511.761401.974441.673874.514163.503060.00032
whereas the real distances (because we knew what the simulation setting was) were
> dist(true.MP$XYs.mat)PoissonNegBinZIPoissZINegBiHurdNBiPoisNBNBPoisOIPoissOINegBiMNegBin4.46711ZIPoiss5.093581.51616ZINegBi5.214221.260690.42784HurdNBi5.224441.196990.523470.09773PoisNB5.108580.895610.813090.425420.33690NBPois5.198831.243480.432080.017980.091390.41228OIPoiss0.001814.468595.095305.215885.226095.110185.20050OINegBi1.695002.853323.738753.768183.759263.587463.751251.69618M4.146882.149421.345871.709591.784551.939701.704934.148683.12059g4.146882.149421.345871.709591.784551.939701.704934.148683.120590.00037
From these matrices, it is readily seen that the real value of *h* in the model projection space was 0.000372 whereas its corresponding estimated *h* value is 0.000323. A quick calculation yields the distances between the true location of the orthogonal projection *M*, its estimate, the true location of *g* and its estimate:
hat.mhat.gtrue.mhat.g0.000323true.m0.3830740.383074true.g0.3830740.3830740.000372
As expected, variation in the quality of these estimates and the difference with the true locations changes from simulated dat set to simulated data set. Two questions are a direct consequence of this observation: first, the MPMS data representation in Figure 5 could be more accurately depicted via bootstrap and confidence clouds or spheres for the location of each model in model space could be drawn. Such task would however involve entertaining the problem of the representation of multiple bootstrap NMDS runs in a single space, using the same rotation.

Classically, variation among NMDS object has been estimated only after Procrustes rotation has oriented the various coordinate systems for maximal similarity among the NMDS objects (see Mardia et al., 1979). A long series of articles involving authors, such as T. M. Cole, S. R. Lele, C. McCulloch, and J. Richtsmeir demonstrates that this approach is deeply flawed. This work is summarized in the monograph by Lele and Richtsmeier (2001). The problem is that the apparent variability among equivalent points in the multiple objects depends on distance from the center of rotation. Lele and Richtsmeir argue that inference is better made regarding variation in estimated distances between points than on the coordinates of points. A mean distance matrix can be estimated from a set of bootstrapped replicates, and it is almost certain that the mean distance matrix will be the most informative matrix both for inference and for graphical purposes as this mean corresponds to the expectation with respect to $\hat{\theta }$ in Akaike’s 5^th^ insight (see section 2.1.5). Further, variation and covariation in all estimated distances and contrasts of distances can be invariantly calculated and used for inference. Finally, extending our MPMS methodology to include confidence bounds for our estimates is a topic of current research in our collaboration and will be treated in a future manuscript because it necessitates the same degree of care used to generate confidence intervals for Model-Average inferences (see for instance Turek, 2013).

The second question has to do with how would our estimate of the location in model space of the orthogonal projection of the generating process compare to the location of the model-average. For our example at hand, the AIC values as well as the ΔAICs were:
> AICsPoissonNegBinZIPoissZINegBiHurdNBiPoisNBNBPoisOIPoissOINegBi14301.3219823.6399327.0868923.3558880.2039168.1299027.72714302.95112625.047
> delta.isPoissonNegBinZIPoissZINegBiHurdNBiPoisNBNBPoisOIPoissOINegBi5421.11814943.43554446.8832843.151460.00000287.92594147.524445422.747693744.84389

**FIGURE 6 |:**
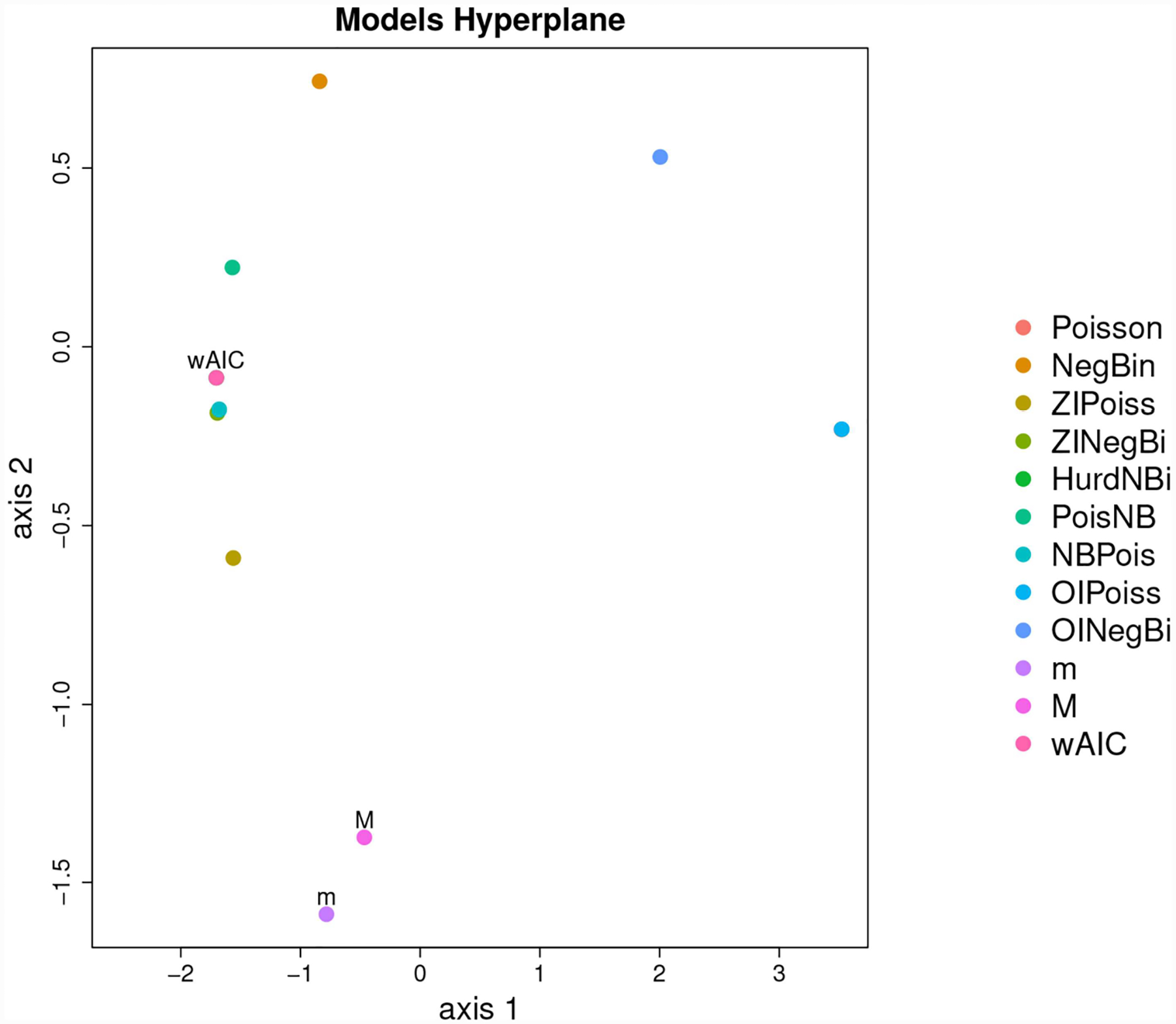
NMDS space of nine models for the Horseshoe crab example (section 3.1). The true projection, *M*, of the generating model onto the NMDS plane is shown, along with the location of the estimated location of such projection, *m*, and of the model average, *wAIC*.

To compare the estimated location of the model average with our estimated our model projection, we plotted both panels in Figure 6 into a single, two-dimensional figure with: the location of every estimated model, the location of the model averaged coordinates using the AIC weights, the location of the estimated orthogonal projection of *g*, and the location of the true location of the orthogonal projection *g*. Such figure is presented in Figure 6. In this figure, the distance between the real projection *M* of *g* and our estimated projection is 0.383074 whereas the distance between the model-average and the real projection of *g* is 1.784555. A quick inspection of Figure 6 shows that this case in fact, is a real-life illustration of the point brought up by Figure 3B. When the geometry of the model space is as in Figures 3B, 5, 6, model averaging may not be a suitable enterprise.

### 3.2. An Ecosystems Ecology Application

**FIGURE 7 |:**
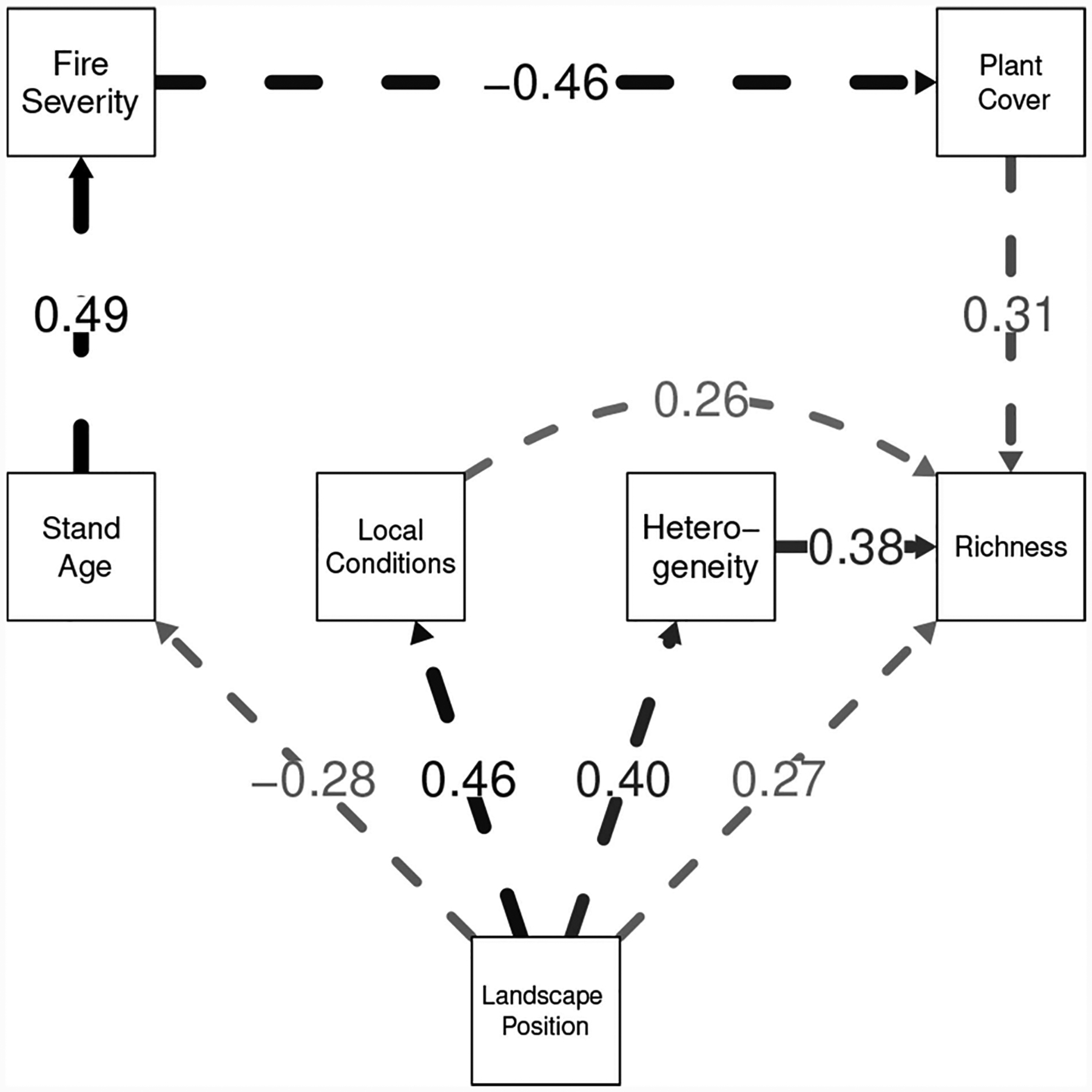
The final, simplified model explaining plant diversity from Grace and Keeley (2006). Arrows indicate causal influences. The standardized coefficients are indicated by path labels and widths. See section 3.2 for details. Prasanta S. Bandyopadhyay, Gordon Brittan Jr., Mark L. Taper, Belief, Evidence, and Uncertainty. Problems of Epistemic Inference, published 2016 Springer International Publisher, reproduced with permission of Springer Nature Customer Service Center.

Here we discuss a worked example highlighting the strengths of the model projections approach to multi-model inference. This example was originally presented in Taper and Ponciano (2016a) which is freely downloadable from: https://link.springer.com/book/10.1007/978-3-319-27772-1 This example is an analysis of data simulated from a structural equation model (SEM) based on a study by Grace and Keeley (2006). Simulation from a known model in necessary to understand how well our methods capture information about the generating process, while basing that model on published research guarantees that our test-bed is not a toy, but is a problem of scientific interest. SEM is a flexible statistical method that allows scientists to analyze the causal relationship among variables and even general theoretical constructs (Grace and Bollen, 2006, 2008; Grace, 2008; Grace et al., 2010). Grace and Keeley (2006) analyzed the development of plant diversity in California shrublands after natural fires. Structural equations models were used to make inferences as to the causal mechanisms influencing changes in diversity. Plant composition at 90 sites was followed for 5 years. The Grace and Keely final model is displayed in Figure 7. To summarize the causal influences, species richness is directly affected by heterogeneity, local abiotic conditions, and plant cover. Heterogeneity and local abiotic conditions are in turn affected by landscape position, but total cover is only directly affected by burn intensity. Burn intensity is in turn only affected by stand age, which itself depend on landscape position. Affects and their direction are shown as arrows in the figure. The strength of affects (i.e., the path coefficients) are shown both as numbers on the figure and as the thickness of the arrows).

Forty-one models were fit to our generated data. The models ranged from underfitted to overfitted approximations of the generating process. The actual generating model was not included in this model set. Using this set of fitted models, we estimated a 2-d Non-Metric Dimensional Scaling model space as discussed above. The calculated stress was tiny (0.006%) indicating almost all higher dimensional structure is captured by an $R^{2}$ plane. A mapping of the estimated space analogous to our Figure 6 is shown in their Figure 6 Taper and Ponciano (2016a). Δ*AIC* values are indicated by color. As in Figure 6 of this paper, on this map of model space we also indicated: (1) The estimated projection (location) of the generating process to the 2-d NMDS space, (2) The Akaike weighted model averaged location and 3) The actual projection of the true generating process l onto the 2-d manifold (in this worked example this can be done because we have simulated from a known model).

Two important observations can be made based on the graph in Figure 6 (both in this manuscript and in Taper and Ponciano, 2016a) : First while there is a rough agreement between proximity to the generating process and Δ*AIC* values, this relationship is not as tight as one might naively expect. The inter-model KL distances do have substantial impact on the map. Second, using our methods and just like in example 3.1 above, the estimated projection of the generating process is somewhat nearer to the actual projection of the generating process than the location produced by model-averaging (Figure 6 in this manuscript).

**FIGURE 8 |:**
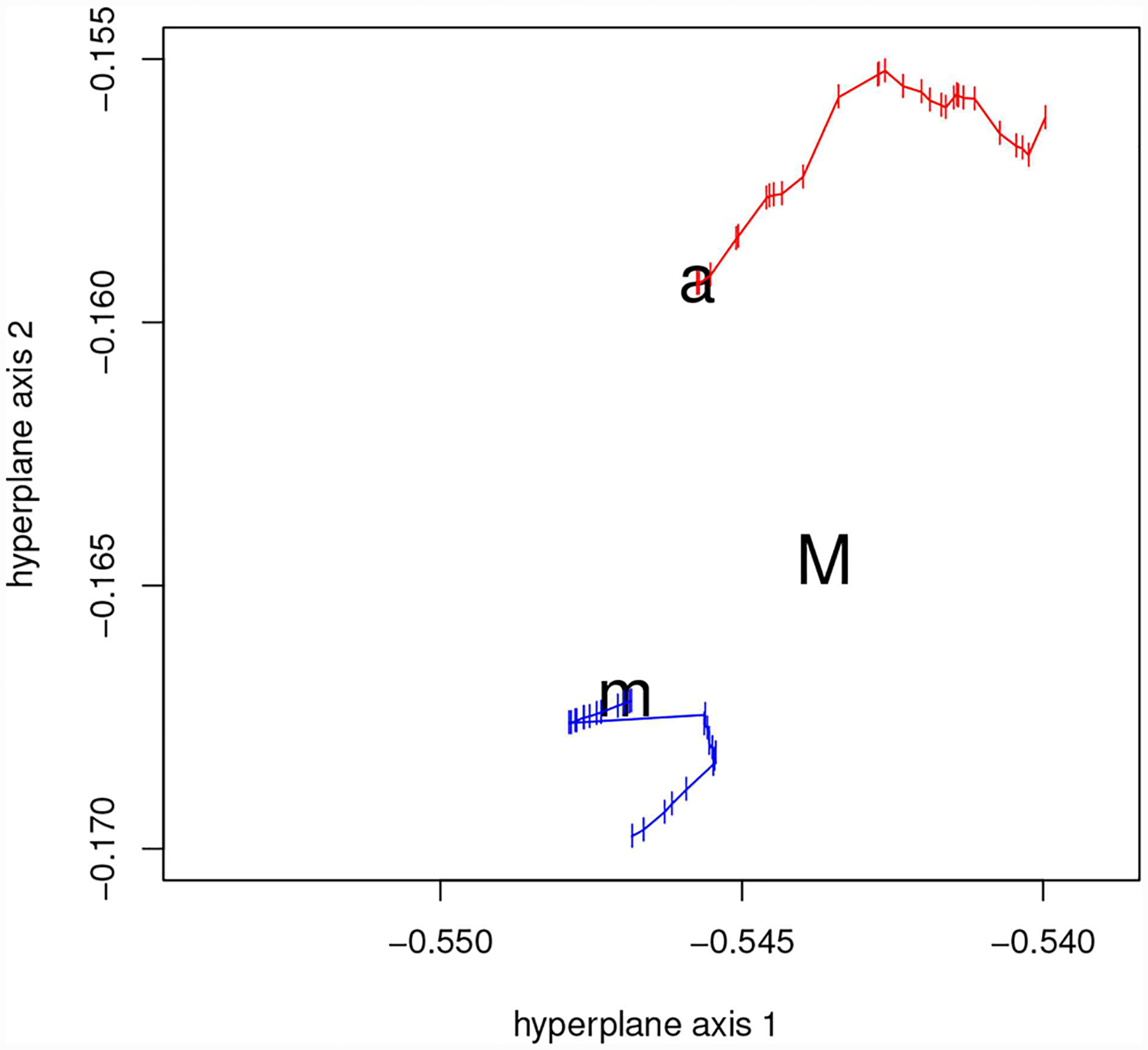
Stability test of the displacement (trajectories) of the model prediction (in blue) and the model average (in red) under deletion of 1 – 30 models. *M* denotes the true location of the orthogonal projection of the generating model in the hyperplane. *m* and *a* mark the location of the model projection and the model average, respectively, when the 30 models are used. In both cases, as models are removed one by one from the candidate model set, the location of both *m* and *a* changes (little vertical lines). Note how the model projection estimate is more stable to changes in the model set than the model average. Prasanta S. Bandyopadhyay, Gordon Brittan Jr., Mark L. Taper, Belief, Evidence, and Uncertainty. Problems of Epistemic Inference, published 2016 Springer International Publisher, reproduced with permission of Springer Nature Customer Service Center.

Figure 8 demonstrates the sensitivities of both the estimated projection and model average of eliminating fitted models from the estimation of the NMDS space. We repeatedly eliminate the left-most model in the model set and reestimate the space after each cycle. With each model elimination, the model-averaged location moves toward the right. On the other hand, the estimated projection stays near its original location, even after all fitted models in that side of the map have been eliminated. Conversely, eliminating from the right, the model average shifts to the left as anticipated. Under right-side model elimination, the model projection is somewhat more variable than under elimination from the other direction.

This model elimination example illuminates differences in the two kinds of estimates the generating process location. These differences follow directly from the geometric development of the AIC by Akaike, and from the mathematics of model averaging. (1) The model average must fall inside of the bounds of the fitted models. changing the model set will, except in contrived cases, change the model average. (2) Because it is a projection, our method’s estimate of the generating process’ location can fall outside the bounds of the model set. And (3), because of the nature of projection geometry, farther models can inform the estimated situation of the generating process in the NMDS map. Point (3) is demonstrated in the discrepancy in the stability of the model projection location under model elimination from the left and model elimination from the right. There are several models with high influence that are deleted quickly under model elimination from the right that stay in the model set much longer under elimination from the left.

Our approach calculates two important diagnostic statistics not even thought of in model averaging. The first is measure of the dispersion of the generating process. This is the neg-selfentropy or *Sgg*. In this example it is calculated to be −9.881, very close to the known magnitude of −9.877. The second statistic is an estimate of the perpendicular distance of the generating process to the NMDS manifold (*h* in Equation 9). This diagnostic is critical for proper interpretation of your model set. If the generating process is far from NMDS manifold, then any statistic based on models in the model set is likely to be inaccurate. Using our approach we calculate *h* to be 0.0002. The known *h* is 6*e* – 08.

### 3.3. Testing the Non-parametric Estimation of *Sgg*

To exemplify the independent estimation of *Sgg* with a data set we simulated samples from a seven-dimensional multivariate normal distribution and compared the true value of *Sgg* with its non-parametric estimate according to Berrett et al. (2019). We chose to simulate data from a multivariate normal distribution because its *Sgg* value is known analytically. When the dimension of a multivariate normal distribution is *p* and is variance-covariance matrix is Σ, then

(12) $$Sgg=-\frac{1}{2}\text{ln}\left\{{\left(2\pi e\right)}^{p}\text{det}(\Sigma )\right\}.$$

**FIGURE 9 |:**
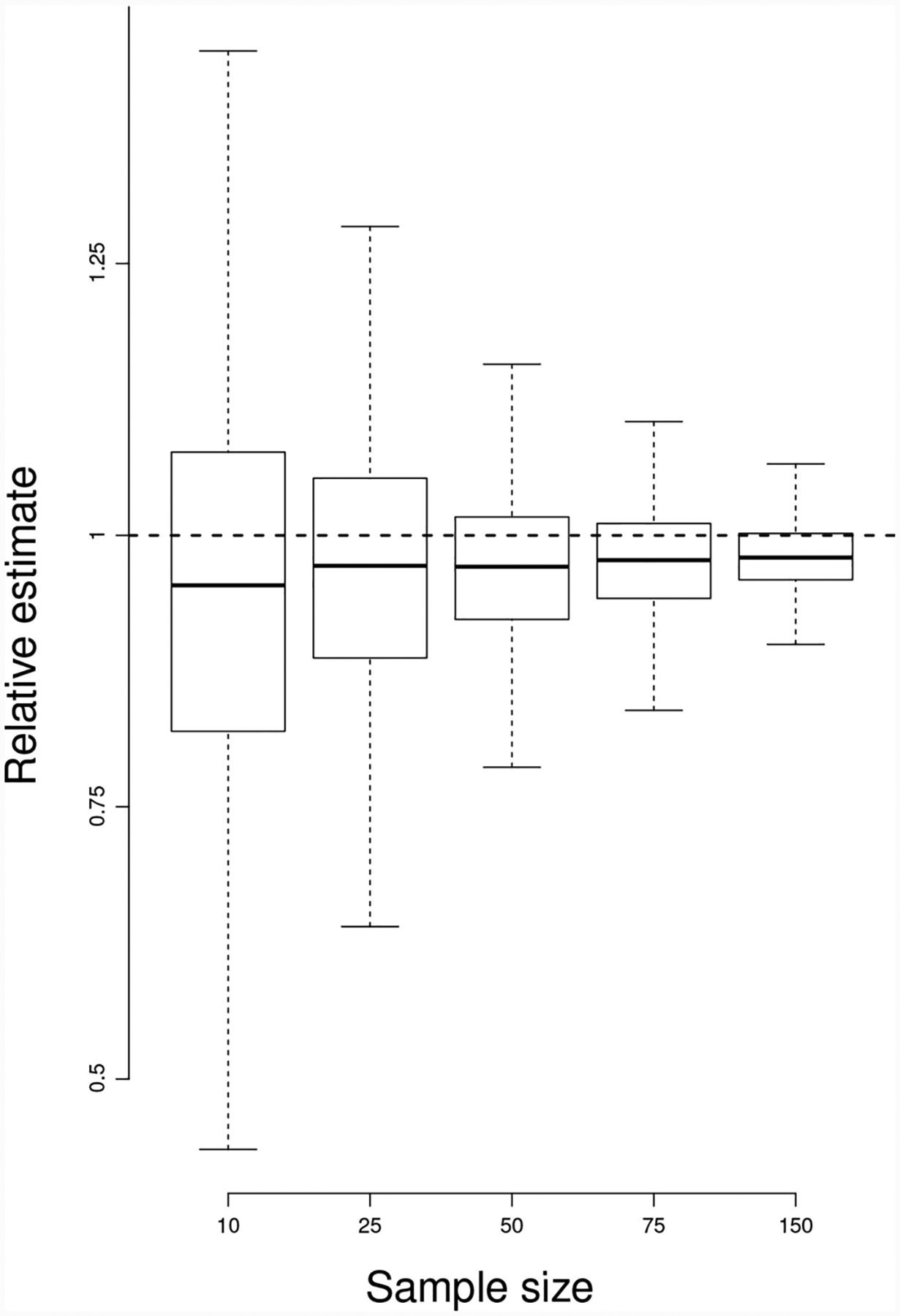
Boxplots of sets of 2,000 non-parametric estimates of *Sgg* (from Berrett et al., 2019) relative to the true *Sgg* value of 9.93257, for different sample sizes. The simulated data comes from a seven-dimensional Multivariate Normal distribution with means equal to 10 and the identity matrix as a variance-covariance matrix. The dashed, horizontal line at 1 shows the zero-bias mark.

To carry our test, we chose five testing sample sizes 10, 25, 50, 75, 150, and for each sample size we simulated 2,000 data sets according to a multivariate normal distribution with *p* = 7 and Σ = *I*, and computed each time Berrett et al.’s non-parametric estimate. The resulting estimates, divided by the true value of 9.93257 are plotted as boxplots in Figure 9.

## 4. DISCUSSION

We have constructed a novel approach to multi-model inference. Standard multi-model selection analyses only estimate the relative, not overall divergences of each model from the generating process. Typically, divergence relationships amongst all of the approximating models are also estimable (dashed lines in Figure 5). We have shown that using both sets of divergences, a model space can be constructed that includes an estimated location for the generating process (the point *g* in Figure 5). The construction of such model space stems directly from a geometrical interpretation of Akaike’s original work.

The approach laid out here has clear and substantial advantages over standard model identification and Bayesian based model averaging. A heuristic approach aiding the development of novel models is now possible by simply being able to visualize a set of candidate models in an Euclidean space. Now the overall architecture of model space vis-a-vis the generating process is statistically estimable. Such architecture is composed of a critical set of quantities and relationships. Among these objects, we now include the estimated coordinates of the closest orthogonal generating model projection onto the manifold of candidate models (the point *M* in Figure 5). Second, the estimated magnitude of the total divergence between the truth and its orthogonal projection onto the manifold of models can give the analyst an indication of whether important model attributes have been overlooked.

In the information criterion literature and all scientific application, the neg-selfentropy *Sgg* of the generating process is simply treated as an unknown quantity. In fact, it can be estimated quite precisely as our example shows. *Sgg* is itself of great interest because with it the overall discrepancy to the generating process becomes estimable. Because this quantity is estimable, now the analyst can discern the overall quality and proximity of the model set under scrutiny. Thus, our approach solves a difficulty that has long been recognized (Spanos, 2010) but yet treated as an open problem.

Studying the model space architecture gives the information to correct for misleading evidence (the probability of observing data that fails to support the best model), accommodation (over-fitting), and cooking your models (Dennis et al., 2019). The scaffolding from which to project the location of the generating process is estimated can be rendered more robust simply by considering more models. This is an interesting result that we expect will later contribute to the discussion of data dredging. On the other hand, non-identifiability and weak estimability (Ponciano et al., 2012) are, of course, still a problem, but at least the model space approach will clearly indicate the difficulties.

As conceived here, model projection is an evidential alternative (Taper and Ponciano, 2016b) to model averaging using Akaike weights (or other Bayesian alternatives) because it incorporates the available information estimated by many models without the redundancy inherent in model averaging. Through model projection the analyst can use more of the information available but usually ignored. Furthermore, our methodology provides new important diagnostic statistics previously not considered by model averaging: *Sgg* and *h*. As we showed in our results, model projection is not as sensitive as model average to the composition of the set of candidate models being investigated. Model averaging appears to artificially favor redundancy of model specification: the more models are developed in any given region of model space, the stronger this particular region gets weighted during the model averaging process. Finally, an emergent pattern in the analysis is that the optimization problem of our model projection methodology can be used to project outside the bounds of the available model set whereas the model averaging methodology, by definition, cannot.

As well as proposing solutions to existing problems, any new method also raises a variety of technical problems that need to be solved. This is certainly the case with the model projection approach presented here.

Our methodology bears a near-model limitation that, although important, is shared with the usage of Akaike’s Information Criterion. Our exposition makes it clear that near model requirement is due to the imperfect yet useful approximation employed by Akaike while setting *ϕ* ≈ *π*/2 (see Figure 1). It was only thanks to this approximation that Akaike was able to solve for the estimable divergence contrasts between all approximating models and the generating process. This approximation breaks down in curved model spaces as the divergence from the generating process increases. Indeed, as the KL distance between approximating models and the generating model increases, −*AIC*/2*n* becomes an increasingly biased and variable estimate of the *Sgf* component of the KL distance between the approximating model and the generating model. This effect is strong enough that sometimes very bad models can have very low Δ*AIC* scores, sometimes even as low as the minimum score. The TIC (Takeuchi, 1976) and the EIC2 (Konishi and Kitagawa, 2008; Kitagawa and Konishi, 2010) are model identification criteria designed to be robust to model misspecification. Substituting one of these information criteria for the AIC in constructing the matrix of inter-model divergences should allow the use of models more distant from truth than is acceptable using the AIC.

Our methodology focuses on estimation of the model space geometry but uncertainties around such estimation are not fully worked out as of yet. Work in progress by Taper, Lele, Ponciano and Dennis, the estimation of the uncertainties associated with doing inference with evidence functions, such as Δ*SIC* scores, can be assessed *via* non-parametric bootstrap techniques. We expect bootstrap to be also useful to reduce the variance of information criterion’s bias correction (Kitagawa and Konishi, 2010).

We think that this model projection methodology should be the starting point to do a careful, science-based inquiry of what are the model attributes that make a model a good model. Knowing the location of the projected best model is an essential component of our multi-model development strategy because a response surface analysis can reveal what model attributes tend to be included near the location of the projected best model thus aiding in the construction of a model closer to the best projection.
